# Financial markets' responses to COVID-19: A comparative analysis

**DOI:** 10.1016/j.heliyon.2022.e10469

**Published:** 2022-09-07

**Authors:** Njamba Kapalu, Odongo Kodongo

**Affiliations:** Wits Business School, 2 St. David's Place Parktown, Johannesburg 2193, South Africa

**Keywords:** Market reaction, Contagion, Covid-19

## Abstract

We examine the effects of covid-19 infections and deaths on bond yields and stock returns and possible contagion effects across markets using data for the period 01.07.2019 through 30.06.2020. Deploying different empirical techniques, we find, unlike some papers in the literature that group countries and run panel regressions, that the effects of covid-19 on the financial markets differed by country depending on the way the outbreak was managed. First, covid-19 *infections* generally had a fleeting effect on stock returns and bond yields. Second, in countries that experienced a covid-19 health crisis during the study period (e.g., UK and Italy), covid-19 *deaths* were associated with bond yield increases. For countries employing less restrictive ways of managing the disease (e.g., Sweden), the covid-19 outbreak elicited investor fears of near-term burgeoning infections and possible concomitant economic effects, which caused bond yields to rise.

## 1 Introduction

Developing capital markets are often regarded as more prone to sudden surges and reversals of capital flows during crisis periods than developed markets (e.g., Sula, 2010). However, recent evidence regarding the “hot money” phenomenon is mixed, with some studies (e.g., Liao and McDowell, 2021) finding that crises precipitate capital flight from developing countries while others (e.g., Forbes and Warnock, 2021) provide evidence suggesting that extreme capital flow movements did not increase during the early part of the covid-19 crisis. Given the conflicting evidence and the fact that some studies indicate that capital flows were an important channel of transmission of effects of the covid-19 crisis on capital markets (e.g., Beirne et al., 2021), it is interesting to ascertain whether covid-19 (infections and deaths) and its attendant management (lockdowns and restrictions) had symmetric effects in developing markets and developed markets. This study sought answers to this question using stock returns and bond yields of emerging and developed markets. Asymmetric reaction of prices to innovations has been attributed, in the literature, to investors' irrational behaviour and investor sentiment (e.g., Pochea et al., 2017) and markets’ core features (Beirne et al., 2021).

**Figure 1:**
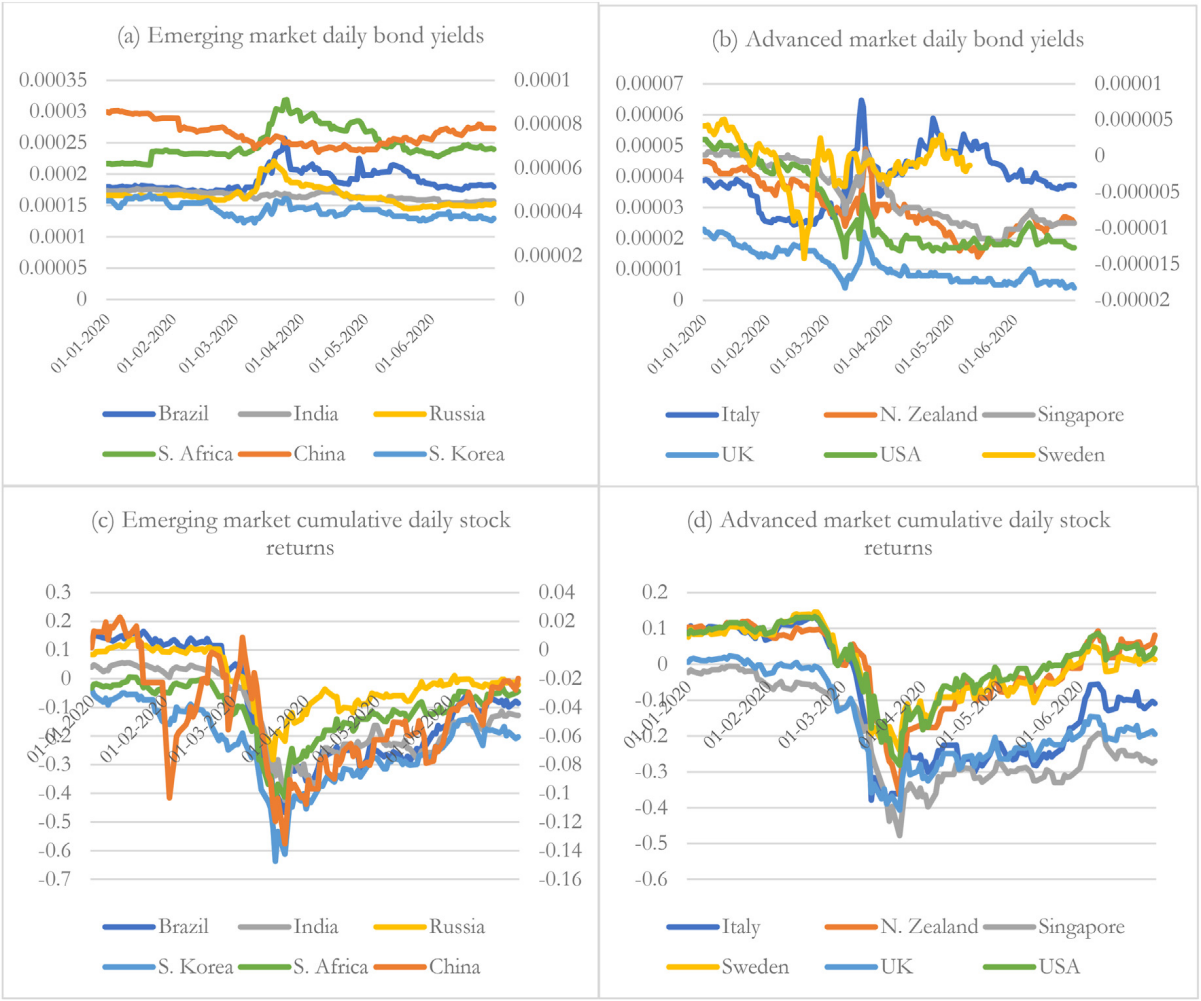
Bond yields and cumulative stock returns during the period January 2020–June 2020. The figure reports cumulative daily bond yields of emerging markets (panel (a)) and developing markets (panel (b)), as well as cumulative daily stock returns of emerging markets (panel (c)) and developing markets (panel (d)) in the sample over the period January 2020 through June 2020.

Stock markets and bond markets generally impound news, and investor sentiment into prices reasonably quickly. Figure 1 shows the behavior of stock cumulative returns and bond yields over the period January 2020 through June 2020. Bond yields generally increase in both markets (panels (a) and (b)) in the second half of March 2020, but advanced market yields (panel (b)) appear more volatile during the period than yields in emerging markets (panel (a)). The apparent differences in bond yield volatility may suggest asymmetric responses of bond prices to the outbreak of the disease across markets. Large and negative response of stock prices across the globe to the covid-19 outbreak, evident in Figure 1 (panels (c) and (d)), is consistent with the empirical evidence of larger magnitudes of reaction that stock markets typically exhibit to negative shocks relative to reactions to positive shocks both in emerging equity markets (e.g., Piveta et al., 2014) and in developed equity markets (e.g., Bird et al., 2017).

However, unlike bond markets, Figure 1 does not appear to suggest that one group of stock markets reacted to the covid-19 outbreak differently from the other (panels (c) and (d)). Notwithstanding this observation, some recent studies have found asymmetric effects of covid-19 in different stock markets (e.g., Topcu and Gulal, 2020). Several reasons motivate the need for a comparative analysis of the effects of the covid-19 crisis in developed and developing markets and the propagation of shocks arising therefrom. First, with increased global trade, there are many thriving and growing stock and bond markets around the world. For instance, BRICS countries (Brazil, Russia, India, China, and South Africa) have been some of the fastest growing economies and therefore have been “major recipients of global investment flows” (Mensi et al., 2014). This raises prospects for possible linkages between BRICS financial markets and financial markets of developed countries.

The figure reports cumulative daily bond yields of emerging markets (panel (a)) and developing markets (panel (b)), as well as cumulative daily stock returns of emerging markets (panel (c)) and developing markets (panel (d)) in the sample over the period January 2020 through June 2020.

Second, to the extent that changes in global economic factors may affect both developed and developing markets, especially given the increased integration of markets (e.g., Boţoc and Anton, 2020), shared external global shocks could be a channel through which fluctuations in the world's economic and financial conditions are transmitted to developing financial markets. Third, in some cases, studies have reported high correlation between emerging and developed stock markets; for instance, Mensi et al. (2014) find that the BRICS countries' stock markets move in tandem with the stock markets of developed countries when markets are bullish but are largely independent in bear markets.

Like the contagious effects of the disease, empirical literature interrogating the effect of covid-19 on the financial markets is fast proliferating. For example, the economic effects of the virus have been likened to those of the 2007/9 global financial crisis (GFC), with the volatility being attributed largely to restrictions on commercial activities, and other disease contagion containment measures (Baker et al., 2020; Sharif et al., 2020). However, Gormsen and Koijen (2020) argue that while the outbreak's effects may spread quicker in the highly integrated environment of the 21^st^ century and cause sharp economic contraction, the real economic consequences are felt with a lag of approximately one to two years.

In their study, Alfaro et al. (2020) find that stock prices of companies in industries exposed to virus transmission (e.g., accommodation, transportation, and entertainment), exhibit the greatest exposure to the crisis and, therefore, witness the highest price declines. Analogously, Mazur, Dang and Vega (2020) find that natural gas, food, healthcare, and software stocks earn high positive returns, whereas equity values in petroleum, real estate, entertainment, and hospitality sectors fall dramatically in the March 2020 US stock market crash triggered by covid-19. Ashraf (2020), in a study of 64 countries, find that stock markets react negatively to growth in covid-19 cases, but the response varies over time depending on the stage of outbreak. Yong and Laing (2020) find significant negative international exposure through foreign trade and foreign assets, but the effect reverses in the long run, whilst Rizvi et al. (2022) document strong evidence of reduction in firm valuations following the outbreak of the disease. In a very interesting study, Sharma et al. (2021) find a strong co-movement between covid-19 confirmed cases (infections) and stock market returns having controlled for exchange rates and temperature.

Studies in developing markets also show that covid-19 adversely affected the performance of stock markets. In a study of six developed and developing equity markets, Gunay (2020) finds that unconditional correlation coefficients increased by around 10% between China and the US, Turkey and the US, Turkey and Italy and Turkey and Spain, with the Turkish stock market emerging as having the strongest co-movement with other countries, indicating its vulnerability to non-systematic risk transmission. Belaid et al. (2021) whose analysis also includes both developed and developing markets document an increase in conditional correlation between emerging and advanced economies, implying growing transmission of uncertainty, during the covid-19 crisis period. For African markets, Takyi and Bentum-Ennin (2021), using daily stock return observations, show that stock market performances fell between 2.7% and 21% during and after the occurrence of covid-19.

For debt markets, Haddad et al. (2020) find that though USA market disruptions were large and comparable to those of the global financial crisis (GFC), there were two differences. Firstly, disruptions appeared within days in comparison to the months it took for the US mortgage crisis to have a similar effect.^1^ However, the disruptions were transient and disappeared just as quickly following the Fed's interventions. Secondly, relative to the GFC whose “disruptions were more pronounced in less liquid asset classes”, investment grade bond prices severely deteriorated due to the covid-19 crisis. Further, monetary policies initially implemented in the US in response to the covid-19-induced economic downturn elicited nearly 100 basis points decline in the yields on 30-year Treasury bonds, which drove up their prices almost 30% (Gormsen and Koijen, 2020). These studies focus on the US debt markets and as such cannot be generalized to emerging debt markets which are typically smaller and more thinly traded.

In the literature, our paper is reasonably close to the recent study of Harjoto et al. (2021), which shows that equity markets across the globe reacted negatively to the covid-19 outbreak, with mortality affecting emerging markets differently from developed markets and the recent work of Beirne et al. (2021), which finds that emerging economies experienced greater effects of the covid-19 crisis than advanced economies and suffered abrupt and substantial capital outflows. Unlike our study which exploits sampled countries’ idiosyncrasies for deeper insights, these two studies pool many emerging and developed markets into two groups to identify cross-market effects, which may mask within-country effects. Second, our paper goes beyond equity markets and provides evidence for developed and emerging bond markets as well.

Our study is also close to Basuony et al. (2021) who, using data that include the covid-19 crisis period, report increased conditional volatilities, which are not symmetric across international stock markets: they find that negative effects of covid deaths were accentuated relative to positive effects of covid recoveries. Their study uses asymmetric EGARCH, allowing them to examine the differential effects of negative and positive shocks on conditional variance and skewness but, unlike our study, does not establish whether developing markets were affected the same way as developed markets or which one of covid infections, management, or death had greater effects on stock returns and bond yields; indeed, their study focuses only on the stock markets.

The outbreak of covid-19 may have impacted many financial markets; however, whether the observed financial markets effects are direct (arising from concerns about the economic impact of the virus and their concomitant effect(s) on investors' portfolio choices) or indirect (e.g., due to contagion from other financial markets) has not been clearly understood.^2^ Therefore, the second purpose of this paper is to establish whether the volatility effects of the virus, if any, may have been transmitted across financial markets and, if so, ascertain potential source markets and destination markets of the contagion. The evidence shows that financial shocks exert a much higher contribution on real economic activity and credit during crisis periods than in normal times (Silvestrini and Zaghini, 2015), motivating the need to understand transmission of shocks across global financial markets during the covid-19 crisis.

The causes of contagion have been generally studied along two dimensions. One involves a shock that has no links to macroeconomic fundamentals and is “solely the behaviour of investors or other financial agents” (Dornbusch et al., 2000). That is, “herding” behavior among investors may induce a “bandwagon effect” in the markets which may elicit similar price reactions, resulting in asset prices across markets deviating from fundamentals (Chiang and Zheng, 2010). The second dimension emphasises volatility spillovers resulting from interdependence among markets (e.g., Zhang et al., 2020). A “significant increase in co-movement during crisis periods” is regarded as evidence of contagion (Baek and Jun, 2011).

The recent study of Amar et al. (2021) provide preliminary evidence of increased spillovers between markets during covid-19 crisis, with developed countries' stock markets showing the tendency to lead developing countries’ stock markets. Further, Akhtaruzzaman et al. (2022) and Kapar et al. (2021) provide evidence of risk spillovers from the United States to four major African and across global stock markets respectively while Fu, Liu, and Wei (2021) show that contagion was stronger for countries with stronger outbreaks. Theoretically, the search for diversification opportunities across markets with low correlation due to reasons such as differences in efficiency levels or low integration (e.g., between advanced and emerging markets) may induce spillovers and contagion. Thus, formal tests need be done to isolate the true contagion effects, if any.

Our study contributes to the literature in several ways. First, we provide, for the first time, evidence of asymmetries in the effects of covid-19 infections, death, and management on developed and emerging capital markets, which covers both bond markets and stock markets. Clarity of differential effects of a crisis of the magnitude of covid-19 is important for the formulation of coordinated policy responses to minimize costs associated with such crises in future. Second, we use different methodologies to demonstrate that differential return and yield effects of covid-19 were robust. Third, we extend the literature on volatility transmission across markets, to show that the observed effects of covid-19 on developing equity returns largely emanated from contagion from advanced markets more than from the various announcements (of infections, deaths, or government policy) in those (developing) countries.

This insight is important since it, for the first time, enables us to correctly attribute the observed return volatility in those markets particularly in the first few months of the covid-19 crisis; and is consistent with the recent findings of Samitas et al. (2022) that contagion was instant following covid-19-induced lockdowns. Further, our results show that covid-19 contagion emanated largely from advanced markets to emerging markets with the US being the leading transmitter of shocks to emerging markets: knowledge of the direction of volatility transmission is important for policy makers to timeously institute measures, such as capital controls, in future crises to hedge local markets against external shocks.

The balance of this paper is organized as follows. Section 2 details the empirical methodologies employed in the analysis. Section 3 describes the data and discusses results of some preliminary analyses. Section 4 presents and discusses the empirical findings. Section 5 concludes.

## 2 Empirical strategy

### 2.1 Event studies

We begin with an event study of major news and policy announcements related to covid-19. This is similar to the approach used by Baker et al. (2020) and Gormsen and Koijen (2020), none of which compares the effects in developed markets with those in developing markets. Our event study uses cumulative returns during the period between 01.01.2020 and 30.06.2020, a subset of our sample period. It should be noted that China recorded some covid-19 cases before our study's commencement date, but cases were more widely reported, and therefore expected to impact markets largely from January 2020 (Gormsen and Koijen, 2020; and Phan and Narayan, 2020). The first event study looks at the announcements pertaining to first covid-19-related infections, and deaths, and first announcements of partial or full lockdowns in the respective countries. Table 1 documents these events dates. The second event study examines the effects of the first lockdown in the European Union, and US policy announcements with potential global ramifications (Table 2).

**Table 1:** Event Study 1: Major covid-19 events.

Country	Stock Index	1st infection announced	1st Death announced	1st lockdown announced
Brazil	Bovespa	26-Feb-2020	18-Mar-2020	24-Mar-2020
China	Shanghai Composite	03-Jan-2020	11-Jan-2020	23-Jan-2020
India	BSE Sensex	30-Jan-2020	13-Mar-2020	25-Mar-2020
Italy	FTSE MIB	31-Jan-2020	23-Feb-2020	22-Feb-2020
New Zealand	NZX	28-Feb-2020	29-Mar-2020	26-Mar-2020
Russia	MOEX	01-Feb-2020	27-Mar-2020	30-Mar-2020
Singapore	SGX	24-Jan-2020	22-Mar-2020	03-Apr-2020
South Africa	JSE Top 40	05-Mar-2020	31-Mar-2020	16-Mar-2020
South Korea	Korea Exchange	20-Jan-2020	21-Feb-2020	n/a
Sweden	Nasdaq Stockholm AB	01-Feb-2020	12-Mar-2020	n/a
United Kingdom	FTSE 100	01-Feb-2020	07-Mar-2020	23-Mar-2020
United States	S&P 500	21-Jan-2020	01-Mar-2020	11-Mar-2020

The major covid-19 events consist of 1st infection reported, 1st death and initial announcement of partial or full lockdown measures. ∗ South Korea and Sweden had not instituted any lockdown measures during the period covered by this study.

**Table 2:** Event Study 2: Material covid-19-related news and policy pronouncements.

Policy pronouncement	Announcement Date
First announcement of lockdown in the Eurozone	22 February 2020
Following the declaration of a national emergency on 13 March 2020, the Fed slashed interest rates by 100 basis points to near zero and further announced large scale asset purchases and launched coordinated swap lines with 5 major foreign central banks	15 March 2020

Eurozone and US policy pronouncements are used rather than sampled country pronouncements, because in most cases, policy pronouncements at the individual country level were made the same day as, or very closely after, the lockdown announcement or amidst worsening infection announcements. For example, countries such as Spain, France, and the UK all instituted lockdown measures and made economic stimulus package announcements within a day of each other, during the period March 10, to March 17, 2020 (Phan and Narayan, 2020). This tendency is observed across countries. Thus, country-level economic policy pronouncements were largely anticipated by the market or where they potentially affected markets, such effects were likely distorted by the confounding effects of lockdown announcements. We use each country's major stock index (see Table 1) and 10-year Treasury bond yields for debt markets.

Typical of event studies, we use average cumulative abnormal returns (ACAR) around the events. The computation is for the window around the event date which we take to be 16 days in total (10 days before the event and 5 days after the event). We employ the traditional single-index model, under which the expected daily return for stock/bond market j on day t is computed as $E\left(R_{jt}\right)={\hat{\alpha }}_{j}+{\hat{\beta }}_{j}R_{mt}$, where ${\hat{\alpha }}_{j}$ and ${\hat{\beta }}_{j}$ are estimated intercept and slope coefficients, respectively; and $R_{mt}$ is the market portfolio return on day t. The market portfolio for the calculation of bond returns is the Vanguard international bond index and the Vanguard emerging market bond index for developed and emerging markets, respectively. For stock returns, the market portfolios are, respectively, MSCI world equity index for developed countries and MSCI emerging markets equity index for emerging markets. Abnormal returns are computed as the difference between the realized rate of return and the expected rate of return, $AR_{jt}=R_{jt}-\;E\left(R_{jt}\right)$; and the cumulative abnormal rate of return is computed by summing the abnormal returns, over time:

(1) $$\text{Cumulative ​abnormal ​return},\;CAR_{jt}=\overset{T_{2}}{\underset{t=T_{1}}{\sum }}AR_{jt}$$

The average cumulative abnormal return for developed and emerging bond and stock markets are then computed as the arithmetic average of the cumulative returns in Eq. (1).

### 2.2 Regression analysis

An important deficiency of the event study is that unlike a natural disaster, a corporate action, or a policy change, which is a single event with the consequences happening after the fact, a disease outbreak leaves in its wake infections and deaths occurring over a long period and does not peak at its beginning (Al-Awadhi et al., 2020; and Ashraf, 2020), so that the event of interest may be confounded, even for short periods, by the continued release of information about the disease and other related developments. Therefore, we perform regression analyses to help identify the effect of covid-19 on the asset markets more robustly. That is, we estimate the international asset pricing model specified in Eq. (2) for each country.

(2) $$r_{t}=\alpha +\beta Covid19_{t-1}+\;\gamma X_{t-1}+\varepsilon_{t}$$

The dependent variable, $r_{t}$, represents stock return/bond yield on day t; α is the intercept whilst the term $Covid19_{t-1}$ represents lagged values of various covid-19 indicators. The vector $X_{t-1}$ represents lagged values of systematic risk factors, defined, in the spirit of Chen et al. (1986), to include equity returns when estimating the bond model, and bond yields when estimating the equity model; spread between yields on ten-year Treasury bonds and three-month Treasury bills (liquidity spread); spread between ten-year Treasury bonds and AAA-rated corporate bonds^3^ of similar maturity (quality spread), change in exchange rates (defined as the dollar price of the local currency following recent studies (e.g., Topcu and Gulal, 2020)), and oil price changes, which some studies have found to experience greater cross-shocks with equity returns, for example, “during the covid-19 crisis than during the period before it” (Salisu et al., 2020).

Inclusion of the exchange rate deserves attention. Studies report immense currency volatility particularly in the early days of the covid-19 crisis. For instance, Hofmann et al. (2021) document extreme exchange rate volatility in emerging markets, sparked by covid-19 outbreak, which resulted in large spreads on their local currency bond markets, while Rai and Garg (2021) provide strong evidence of volatility spillovers between stock and currency markets in the BRIICS economies (BRIICS includes Indonesia), especially during the initial days of lockdowns. It is, therefore, necessary to control for exchange rate changes. We use the generalised method of moments (GMM) to control for endogeneity and calculate Newey-West heteroscedasticity and autocorrelation-robust standard errors. The instrument variables used include all the independent variables in their undemeaned, unscaled form (Kodongo and Ojah, 2011), an additional variable, returns on Bitcoin, which has been mentioned as an alternative investment during the covid crisis^4^, and a vector of ones. Eq. (2) is estimated alternately with data for the period 01.07.2019 through 30.06.2020 and with each of the following covid-19 indicators.

1. Daily covid-19 deaths, normalized by the country's population.
2. Daily covid-19 infections, normalized by the country's population.
3. A dummy variable taking the value of “1” after the first infection is recorded and “0” elsewhere.

### 2.3 Contagion

To measure market integration, we begin by calculating the conditional correlation. We employ the vector autoregressive model for estimating the conditional mean of returns. The estimated mean equation follows the specification in Ling and Gurjeet (2010):

(3) $$r_{t\;}=c+\varphi_{t-1}r_{t-1}+\varepsilon_{t}+\theta \varepsilon_{t-1},\varepsilon_{t}|\Omega_{t-1}∼N\left(0,H_{t}\right)$$

where $r_{t\;}$ is a vector of asset returns, c is a vector of constants, $\varepsilon_{t\;}$ is an error terms vector, $\Omega_{t-1}$ is the information set available at time t−1; and $H_{t}\;$ is the conditional variance-covariance matrix of asset returns. The conditional covariance matrix, $H_{t}$, is estimated using the multivariate BEKK-GARCH model proposed by Engle and Kroner (1995), chosen due to its parsimony. The model is specified in Eq. (4).

(4) $$H_{t}=C+A^{\prime }\left(\varepsilon_{t-1}\varepsilon_{t-1}^{\prime }\right)A+B^{\prime }H_{t-1}B$$

where C, A and B are k×k matrices of parameters, and C, the constant matrix, is symmetric. The existence of high conditional correlation provides evidence of integration and, hence, possible financial contagion. High ARCH effect confirms the existence of spillover effects while large GARCH effects represent the persistence of volatility, denoting that the effects of today's shocks remain for many periods in future.

## 3 Data and preliminary analysis

### 3.1 Sampling and data

We represent advanced markets by Italy, New Zealand, Singapore, Sweden, the UK, and USA. Choice of these countries is informed by the initial effects and management of covid-19 and, in some cases, importance in the capital markets. To illustrate, USA experienced one of the worst outbreaks in the world by number of infections and deaths. It is also central to global capital flows: UNCTAD data show that in 2019, USA was the largest recipient of FDI flows and was also a major FDI source country; thus, it would assist us to understand if the central role that it plays in global capital flows may have enabled the transfer of shocks to asset prices in other countries following covid-19's initial effect. Italy and the UK take on an elevated significance because they were the initial epicentres of covid-19 outbreak in Europe. Of the developed markets in our sample, Sweden initially ignored the lockdown strategy of containing the spread of infections hoping to achieve herd immunity through natural exposure, making it interesting to establish whether its capital asset prices experienced similar effects as other countries. To properly identify the effect of covid-19 on the capital markets, we include two developed countries in the South, Singapore, and New Zealand, which appear not to have been as severely affected as those in the North.

Developing markets are represented by BRICS (Brazil, Russia, India, China, and South Africa). We choose BRICS markets mainly because they are “major recipients of global investments and capital flows” (Mensi et al., 2014). Secondly, the BRICS bloc bestrides the bulk of developing regions, representing Asia, Africa, and Latin America. Though not exhaustive by any measure, this choice should therefore give us insights into the possible effects of contagion across continents. Further, Brazil, India, and South Africa were the most affected countries, by number of infections and deaths, in their respective regions during our study period whilst China was the world's epicentre of the virus. Further, we include South Korea, touted as a successful case in managing the virus through quick and early nationwide testing.

For equity markets, we use stock indices, namely, S&P 500 (US), FTSE 100 (UK), FTSE MIB (Italy), Nasdaq Stockholm AB (Sweden), SGX (Singapore), NZX (New Zealand), JSE Top 40 (SA), Bovespa (Brazil), S&P BSE Sensex (India), Shanghai Composite (China), the MOEX (Russia) and the Korea Exchange Index (South Korea). With regards to the oil price, we use the WTI Crude oil prices. To get a consistent sample, where there is more than one stock market in a country, we use only the major stock market. For debt markets, we use yields on 10-year Treasury bonds of all sampled countries. The 10-year bond is believed to exhibit greater investor preference than bonds with longer maturities such as 30-years (Jones, 2000) and experience greater volatility than bonds of shorter maturities (Balduzzi et al., 2001). The greater volatility makes the 10-year bond suitable because the study relies on price movements to identify the effects of covid-19.

The complete dataset covers the period 01.07.2019 through 30.06.2020. The year 2019 had been good for stocks and bonds^5^ and the inclusion of the 6-month period prior to the covid-19 outbreak enables us to achieve quasi-experiment-like conditions for a more robust identification of the effects of the covid-19 crisis on asset returns. The first half of 2020 includes the first wave of the virus, the novel period of economic lockdowns, and the period with most of the macroeconomic policy responses that may have impacted the sampled markets. Table 3 shows the date of the first documented case of covid-19 infections in our sample.

**Table 3:** Date of first covid-19 case.

Country	Stock index	Day of first covid-19 confirmed case in 2020
Brazil	BOVESPA	26-Feb-2020
China	Shanghai Composite	03-Jan-2020
India	BSE Sensex	30-Jan-2020
Italy	FTSE MIB	31-Jan-2020
New Zealand	NZX	28-Feb-2020
Russia	MOEX	01-Feb-2020
Singapore	SGX	24-Jan-2020
South Africa	JSE Top 40	05-Mar-2020
South Korea	Korea Exchange	20-Jan-2020
Sweden	Nasdaq Stockholm AB	01-Feb-2020
United Kingdom	FTSE 100	01-Feb-2020
United States	S&P 500	21-Jan-2020

Data are observed on a daily frequency. The data therefore include daily covid-19 deaths/infections as well as daily stock returns and bond yields. Daily frequency is deemed the most appropriate for isolating volatility effects of announcements in markets with good levels of information efficiency. Stock prices, Treasury bill yields, Treasury bond yields, and exchange rate data are drawn from *EquityRT*, Yahoo! Finance, and FRED databases. Covid-19 data are from the European Centre for Disease Prevention and Control. Stock, oil, and exchange rate returns are computed as $r=\ln\;P_{t}–\ln\;P_{t-1}$, where ln(·) is the natural logarithm and r is the rate of return. For Treasury bonds and bills, the nominal daily yield is obtained as $r={\left(1+k\right)}^{1/365}-1$, where k is the annualized yield. The returns/yields are thereafter mean-centred and are scaled by each variable's own standard deviation.

### 3.2 Descriptive analysis

#### 3.2.1 Stock returns

Table 4 presents a summary of the stock index returns and their autocorrelations for each market. Panel (a) reports summary statistics: New Zealand had the highest mean daily return over the study period (0.03%) while Singapore reports the lowest mean daily return (−0.10%). This may reflect the stock market reaction during the period. Singapore's economy was hard hit by both supply-side and demand-side shocks with sectors like wholesale and manufacturing, which form a large part of the economy, suffering serious supply chain disruptions^6^. For New Zealand, however, their ability to “crush the curve”^7^ by June 2020 could have inspired greater investor confidence, resulting in higher mean returns. All countries' index returns exhibit negative skewness and excess kurtosis, therefore, strongly rejecting the hypothesis of normality as the Jarque-Bera statistics show. For stock returns, the observation of non-normality is not unusual and should not be a major concern for empirical tests provided that residuals exhibit normality (Brooks, 2014).

**Table 4:** Descriptive statistics and autocorrelations for stock returns.

		Mean		Std Dev		Skewness		Kurtosis		Jarque-Bera	
Panel (a): Summary statistics											
Brazil		-0.0003		0.0271		-1.5483		15.4276		17845^S^	
China		-0.0001		0.0107		-1.9118		15.5337		18670^S^	
India		-0.0005		0.0197		-1.5377		16.5384		2096^S^	
Italy		-0.0004		0.0214		-3.4734		33.3156		10519^S^	
New Zealand		0.0003		0.0168		-0.2016		13.0682		1104^S^	
Russia		-0.0001		0.0154		-0.9556		13.6190		1266^S^	
Singapore		-0.0010		0.0153		-0.7416		10.2893		602^S^	
South Africa		-0.0002		0.0183		-1.0541		10.9620		738^S^	
South Korea		-0.0008		0.0218		-0.0599		9.8270		507^S^	
Sweden		0.0001		0.0176		-1.4375		11.8336		938^S^	
UK		-0.0008		0.0172		-1.3030		13.9659		1382^S^	
USA		0.0002		0.0210		-0.8839		13.0619		1135^S^	
Panel (b): Autocorrelations											
Lag	1		2		3	4	5		7		14
Brazil	-0.268^a^		0.181^a^		0.043	-0.021	0.137^a^		0.272^a^		0.129^a^
China	-0.078		0.055		0.061	-0.108^a^	0.034		0.045		0.046
India	-0.108^a^		0.032		0.049	-0.029	0.226^a^		0.145^a^		0.021
Italy	-0.068		0.249^a^		0.098	0.051	0.077		0.097		0.050
New Zealand	0.110^a^		0.202^a^		0.007	0.045	-0.017		0.022		0.006
Russia	-0.047		0.153^a^		0.078	0.194^a^	-0.088		0.091		-0.046
Singapore	-0.010		0.198^a^		0.119^a^	-0.064	0.227^a^		-0.006		0.091
South Africa	-0.077		0.095		0.140	0.035	0.074		0.163^a^		0.178^a^
South Korea	-0.094		0.186^a^		0.114^a^	-0.043	0.033		-0.047		0.086
Sweden	-0.040		-0.012		0.023	0.046	0.086		0.194^a^		0.140^a^
UK	-0.015		0.039		-0.007	0.059	0.068		0.217^a^		0.166^a^
USA	-0.357^a^		0.266^a^		-0.030	-0.105	0.144^a^		0.372^a^		0.225^a^

This table reports summary statistics of stock returns in the named markets during the period 01.07.2019 through 30.06.2020. ^S^ indicates that the reported Jarque-Bera statistic is statistically significant, and ^a^ indicates that the autocorrelation is significantly different from zero. Std Dev represents standard deviation of returns.

Panel (b) reports the autocorrelation outputs. Significant autocorrelation of returns indicates possible return predictability. The results show that return autocorrelations are generally negative at the first lag but turn (generally) positive subsequently. A plausible explanation of the negative lag 1 autocorrelation is that inadequate information and increased uncertainty during the covid-19 period may have induced panic “herd behavior” in which investors collectively (over)react to negative news (say about new infections) by taking short positions and to positive news (say, announcement of an economic stimulus) by taking long positions. In subsequent trades, the markets appear to recover from the initial overreaction (positive autocorrelations in lags 2 and higher). The table shows significant positive autocorrelation for many countries at lags 2 and 7, indicating possible weekly (lag 7) return predictability or possible irrational “positive feedback loops” reminiscent of crisis periods (Kim and Wei, 2002).

The correlation matrix for stock returns is reported in Table 5. As seen in the table, the correlation between pairs of markets was high, especially in advanced markets: for example, above 80% correlation is recorded between the UK and Sweden, Italy and the UK, and Italy and Sweden. Singapore and South Korea, markets in the same region, also report a correlation coefficient above 80%. In general, correlations are high and positive, suggesting either possible contagion across markets, possible similarity of the effects of the crisis across markets, or proximity effects of geographically neighboring markets.

**Table 5:** Stock returns correlation matrix.

	BRA	CHI	IND	ITA	NZE	RUS	SGP	ZAF	SOK	SWD	UKI
China	0.27										
India	0.48	0.45									
Italy	0.67	0.28	0.51								
New Zealand	0.41	0.33	0.46	0.48							
Russia	0.41	0.23	0.40	0.62	0.44						
Singapore	0.49	0.57	0.71	0.53	0.58	0.35					
South Africa	0.63	0.47	0.58	0.71	0.59	0.61	0.68				
South Korea	0.34	0.54	0.58	0.37	0.48	0.26	0.82	0.60			
Sweden	0.65	0.36	0.50	0.84	0.48	0.62	0.59	0.74	0.41		
UK	0.69	0.35	0.54	0.84	0.52	0.68	0.58	0.78	0.46	0.88	
USA	0.79	0.27	0.39	0.69	0.38	0.42	0.43	0.64	0.28	0.70	0.74

The table reports correlations between returns on pairs of stock markets. BRA represents Brazil, CHI is China, IND is India, ITA is Italy, NZE is New Zealand, RUS is Russia, SGP is Singapore, ZAF is South Africa, SOK is South Korea, SWE is Sweden, and UKI is United Kingdom.

#### 3.2.2 Bond yields

Table 6 presents a summary of daily yields for each of the sampled market's 10-year government bonds. Daily bond yields are, on average, below half a percentage point with very minimal standard deviations, which is expected given that, theoretically, government bonds have almost zilch default risk so that volatility largely represents interest rate fluctuations, also expected to be very low on a day-to-day basis (panel (a)). Like in the case of stock returns, the hypothesis of normality is largely rejected in bond yields. Given the low risk inherent in bonds, yields are likely to be predictable over short intervals of time, which explains the high and persistent autocorrelations (Panel (b)). The correlation matrix for bond yields is shown in Table 7. High correlations are reported between many markets with Singapore recording high correlations with most markets except Brazil, Italy, and South Africa. We also document high correlations between USA and China, India, Singapore, New Zealand, and the UK and a strong negative correlation with South Africa.

**Table 6:** Descriptive statistics and autocorrelations for bond yields.

		Mean		Std Dev		Skewness		Kurtosis		Jarque-Bera	
Panel (a): Summary statistics											
Brazil		0.00019		0.00001		1.7234		7.7337		374^S^	
China		0.00008		0.00001		-0.5671		1.9049		27^S^	
India		0.00017		0.00001		-0.7858		2.7510		27^S^	
Italy		0.00004		0.00001		0.2536		2.6591		4	
New Zealand		0.00003		0.00001		-0.2989		2.3031		9^S^	
Russia		0.00017		0.00002		0.2597		2.5473		5^S^	
Singapore		0.00004		0.00001		-0.8879		2.3040		39^S^	
South Africa		0.00023		0.00002		1.6193		4.8573		152^S^	
South Korea		0.00004		0.00000		0.0431		2.2632		6^S^	
Sweden		0.00000		0.00000		-0.6640		2.7216		20^S^	
UK		0.00001		0.00001		-0.2787		1.7773		20^S^	
USA		0.00004		0.00001		-0.5192		1.6330		32^S^	
Panel (b): Autocorrelations											
Lag	1		2		3	4	5		7		14
Brazil	0.936^a^		0.868^a^		0.813^a^	0.744^a^	0.689^a^		0.591^a^		0.321^a^
China	0.989^a^		0.978^a^		0.970^a^	0.962^a^	0.953^a^		0.934^a^		0.870^a^
India	0.964^a^		0.934^a^		0.903^a^	0.870^a^	0.836^a^		0.808^a^		0.685^a^
Italy	0.955^a^		0.901^a^		0.863^a^	0.823^a^	0.788^a^		0.701^a^		0.528^a^
New Zealand	0.968^a^		0.930^a^		0.903^a^	0.885^a^	0.865^a^		0.801^a^		0.643^a^
Russia	0.981^a^		0.954^a^		0.921^a^	0.888^a^	0.849^a^		0.772^a^		0.573^a^
Singapore	0.984^a^		0.966^a^		0.951^a^	0.933^a^	0.917^a^		0.881^a^		0.788^a^
South Africa	0.986^a^		0.970^a^		0.951^a^	0.928^a^	0.904^a^		0.850^a^		0.685^a^
South Korea	0.960^a^		0.924^a^		0.893^a^	0.864^a^	0.836^a^		0.773^a^		0.521^a^
Sweden	0.964^a^		0.919^a^		0.874^a^	0.820^a^	0.767^a^		0.671^a^		0.414^a^
UK	0.965^a^		0.929^a^		0.897^a^	0.865^a^	0.830^a^		0.770^a^		0.647^a^
USA	0.986^a^		0.970^a^		0.957^a^	0.945^a^	0.932^a^		0.899^a^		0.807^a^

This table reports summary statistics of bond yields in the named markets during the period 01.07.2019 through 30.06.2020. ^S^ indicates that the reported Jarque-Bera statistic is statistically significant at the 10% level or better.

a indicates that the autocorrelation is significantly different from zero, and Std Dev represents standard deviation.

**Table 7:** Correlation matrix for bond yields.

	BRA	CHI	IND	ITA	NZE	RUS	SGP	ZAF	SOK	SWD	UKI
China	-0.47										
India	-0.19	0.68									
Italy	0.52	-0.42	-0.41								
N. Zealand	-0.30	0.72	0.66	-0.18							
Russia	0.42	0.23	0.50	0.08	0.36						
Singapore	-0.24	0.81	0.83	-0.38	0.85	0.60					
S. Africa	0.62	-0.83	-0.49	0.47	-0.46	-0.02	-0.59				
S. Korea	-0.22	0.37	0.36	-0.06	0.56	-0.13	0.36	-0.02			
Sweden	-0.13	0.15	-0.06	0.30	0.44	-0.37	0.03	0.04	0.67		
UK	-0.30	0.81	0.77	-0.24	0.91	0.36	0.89	-0.55	0.61	0.37	
USA	-0.39	0.91	0.80	-0.37	0.84	0.40	0.94	-0.76	0.40	0.17	0.92

The table reports correlations between returns on pairs of bond yields of markets. BRA represents Brazil, CHI is China, IND is India, ITA is Italy, NZE is New Zealand, RUS is Russia, SGP is Singapore, ZAF is South Africa, SOK is South Korea, SWE is Sweden, and UKI is United Kingdom.

**Figure 2:**
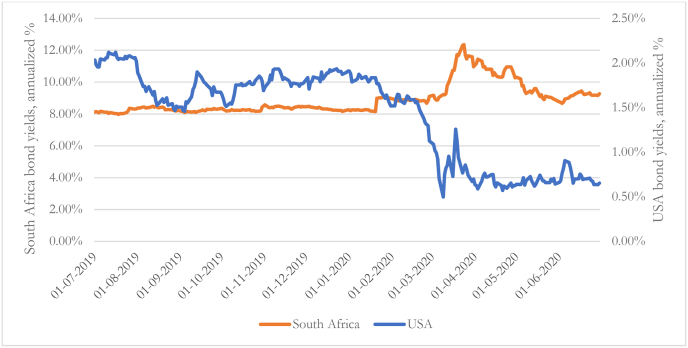
Bond yields for USA and South Africa, 07:2019–06:2020.

The high negative correlation with South Africa could potentially indicate capital flight from the relatively weak South Africa's economy during the covid-19 crisis. As shown in Figure 2, bond yields declined sharply in the US market between January and April 2020 due, perhaps, to high demand as investors sought “safer havens” to shield themselves from the covid-19-related volatility; they were rising in South Africa, reflecting possible flight of capital away from the perceived high-risk market. Additional to the shock introduced by covid-19, South Africa suffered a sovereign credit downgrade towards the end of March 2020, by Moody's, which had issued a downgrade warning early February 2020. Both the warning and the subsequent action may have signalled investors about the country's falling credit quality and induced capital flight.^8^

## 4 Empirical tests results

### 4.1 Results of event studies

We now report results of each market's reaction to various aspects of the covid-19 outbreak and related announcements. In the first set of studies (Event Study 1), three events are considered: the date of first infection, the date of first death, and the first lockdown announcement in each country. The specific dates for each market are tabulated in Table 1. In the second set of studies (Event Study 2), we examine whether two specific events, the first announcement of lockdown within the Eurozone, specifically in Italy on the 22 February 2020; and the slashing of interest rates by the US Federal Reserve (the Fed) on 15 March 2020, affected each of our sampled markets.

#### 4.1.1 Event study 1: emerging markets

**Figure 3:**
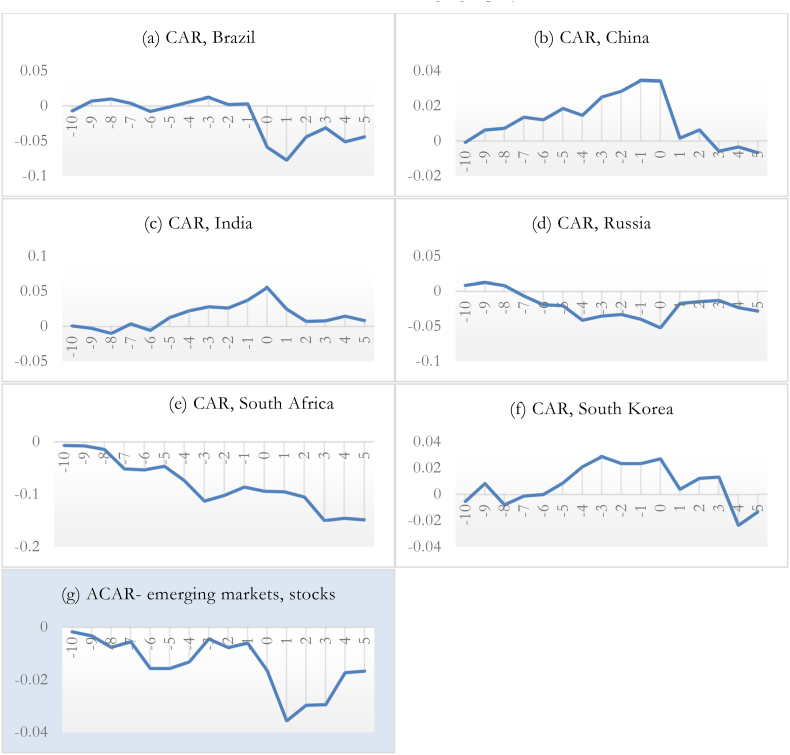
CAR and ACAR for first COVID-19 infection in emerging equity markets. This figure reports cumulative abnormal equity returns (CAR) for Brazil (panel (a)), China (panel (b)), India (panel (c)), Russia (panel (d)), South Africa (panel (e)) and South Korea (panel (f)) as well as average cumulative abnormal equity returns (ACAR) for all the six emerging markets (panel (g)). We use daily covid-19 infections data for the period January 2020 through June 2020.

**Figure 4:**
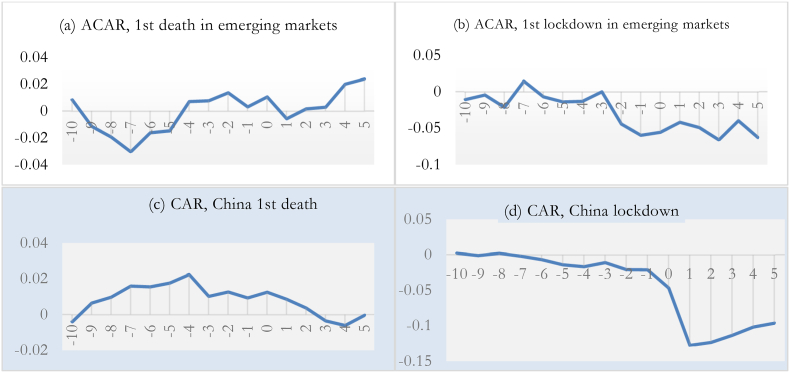
Emerging market equity returns around death and lockdown announcements. In this figure, we present plots of average cumulative abnormal equity returns associated with covid-19 deaths (panel (a)) and for the first lockdown (panel (b)) in emerging markets, and cumulative abnormal equity returns associated with the first death (panel (c)) and lockdown (panel (d)) in China. We use daily data for the period 01:2020 to 06:2020.

The results of event studies for emerging equity markets are presented in Figure 3 (announcement of first infection) and Figure 4 (announcement of first death and lockdown). We report CAR for each country and ACAR for the six markets separately. Both CAR and ACAR are calculated for an event window of 16 days (−10, 0, 5), the “tight” window being chosen to lessen potential confounding effects. Consistent with Aizenman et al. (2016) who find negative effects on stock returns as a result of the global financial crisis, we find that all emerging markets had their stock returns affected by covid-19. Except for Russia (panel (d)), where the announcement appears to trigger an upward revision in stock returns, news of the first covid-19 infection appears to depress stock markets in all the emerging equity markets. The rout is pronounced for China (panel (b)), India (panel (c)), South Africa (panel (e)), and South Korea (panel (f)), but mild for Brazil (panel (a)). For the emerging markets combined (the event study test, panel (g)), the average cumulative abnormal equity returns fall sharply on announcement although a mild decline appears to begin a day earlier. The latter finding can potentially be attributed to news leakages about the first infection ahead of the official announcement.

The ACAR results for emerging stock returns around the days of announcement of the first death and the first lockdown are presented in Figure 4. There were no lockdowns in South Korea over the period of study, so panel (b) has only five markets averaged. The results are mixed: for first death (panel (a)), a sharp decline in stock returns is evident on the day of announcement, which is followed by a sustained increase in returns; for lockdown announcements (panel (b)), however, a sharp decline in abnormal returns is observed three days before the announcement, probably because the lockdown announcement was closely preceded by a related announcement such as first death (e.g., India) or first infection (e.g., South Africa): under such circumstances, event studies may yield unreliable results and, so it is important to perform additional tests to identify a more robust relationship. We address this concern in Section 4.2 using regression analysis.

Contrary to the mixed signals in emerging markets as a group, Chinese CAR on stocks (Figure 4) shows clear evidence of a downward shock on each of the days of announcement of first death (panel (c)) and first lockdown (panel (d)) measures. This is expected since China was always the first to make such announcements as the disease's global epicentre; in the case of the lockdown, however, the slide appears to have started a few days earlier, again due, perhaps, to news leakages or related announcements.

**Figure 5:**
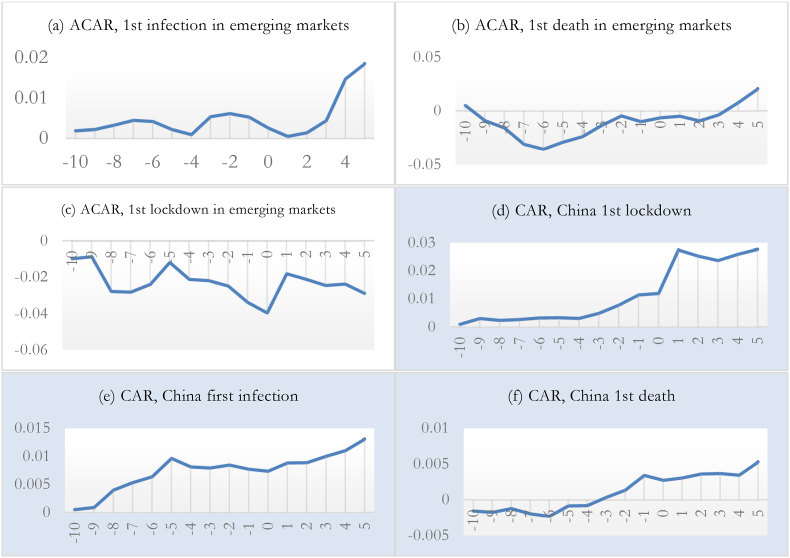
Emerging markets bond yields around infection, death, and lockdown announcements. The figure shows plots of average cumulative abnormal bond yields for the first infection (panel (a)), first death (panel (b)) and first lockdown announcement (panel (c)) in emerging markets, and cumulative abnormal bond yields for China relating respectively to first lockdown announcement (panel (d)), first infection (panel (e)) and first death (panel (f)) following the covid-19 outbreak. We use daily data for the period January 2020 through June 2020.

Next, we conduct Event Study #1 on bond yields. Our interest in bond yields is motivated by the many studies that have established a nexus between the term structure and macroeconomic fundamentals such as inflation and output growth (e.g., Chun, 2011) and business cycles (Kumar et al., 2021). Therefore, understanding the effect of covid-19 on bond yields is a first step towards appreciating its effects on the real economy. Figure 5 shows results of the event studies for emerging markets’ bond yields. The results show a strong upward adjustment in bond yields on the day of lockdown announcement (panel (c)), a possible indication of investor jitters about the implications of the development to the economy; it may also indicate the “flight-to-safety” phenomenon, as investors rush to rebalance their portfolios to allocate more or all of their investments to safer assets such as gold or to markets perceived to represent less vulnerability (e.g., developed bond markets). However, the sharp increase in yields is not persistent.

The first infection announcement (panel (a)) triggers a mild decline in bond yields on the announcement day, which is followed by a sharp and persistent increase in yield over subsequent days; similarly, the announcement of first death (panel (b)) triggers substantial yield increases in emerging markets over several days, both consistent with the findings of Andries et al. (2020) that higher levels of sovereign bonds uncertainty are associated with covid-19 infections and containment measures. We compare the developments in emerging markets to the case of China. The results show that the Chinese bond market reacted adversely to all the three announcements, with the lockdown (panel (d)) appearing to take the greatest toll on bond prices. Unlike announcements of infections and deaths (panels (e) and (f)), which largely generate health concerns, and, for China, happening when the world was only just getting familiar with the devastating consequences of the disease, the lockdown policy had direct economic implications: manufacturing would pause with serious consequences to industrial production; the service sector, except firms with significant online presence, would take a break, and the informal sector, the would-be last resort of newly-unemployed labor, would grind to a halt, all of which would be expected to put a strain on the economy. Bond markets were therefore wont to respond sharply to lockdown announcements.

#### 4.1.2 Event study 1: developed markets

**Figure 6:**
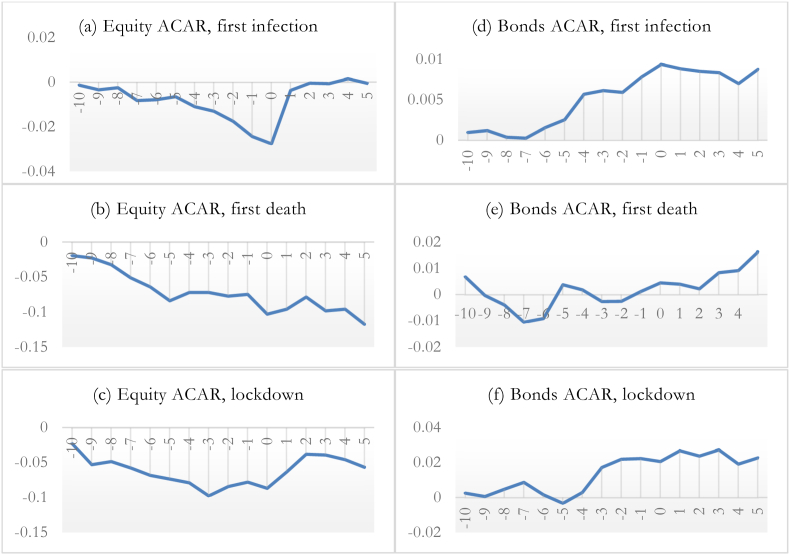
Developed markets equity returns and bond yields around infection, death, and lockdown announcements. The figure shows plots of average cumulative abnormal equity returns and bond yields for the first infection (panels (a) and (d)), first death (panels (b) and (e)) and first lockdown announcement (panels (c) and (f)) following covid-19 outbreaks in developed markets. We use daily data for the period January 2020 through June 2020.

The average cumulative abnormal returns for developed markets, are plotted in Figure 6 for first death (panels (a) and (d)), first infection (panels (b) and (e)), and first lockdown (panels (c) and (f)) announcements both for equities and bonds. The ACAR calculations for bonds and stocks for developed markets exclude Sweden with regards to the lockdown since Sweden, like South Korea, did not institute any lockdown measures during the period covered by this study. Unlike emerging markets, where we find strong and unequivocal evidence of the covid-19 effects, our evidence for developed markets appears mixed and inconclusive. For instance, equity returns increase substantially while bond yields fall marginally on news of first infection in advanced markets, and on news of first death. However, consistent with intuition, bond yields increase on the lockdown announcement. Overall, the mixed reactions observed with the bond yields, and equity abnormal returns, suggest that a study to more accurately isolate the effects of covid-19 on the financial markets is important. This motivates the need for the regression analysis, which directly controls possible confounding effects of other variables, and the examination of volatility spillovers.

#### 4.1.3 Event study 2: material covid-19-related news and policy announcements

**Figure 7:**
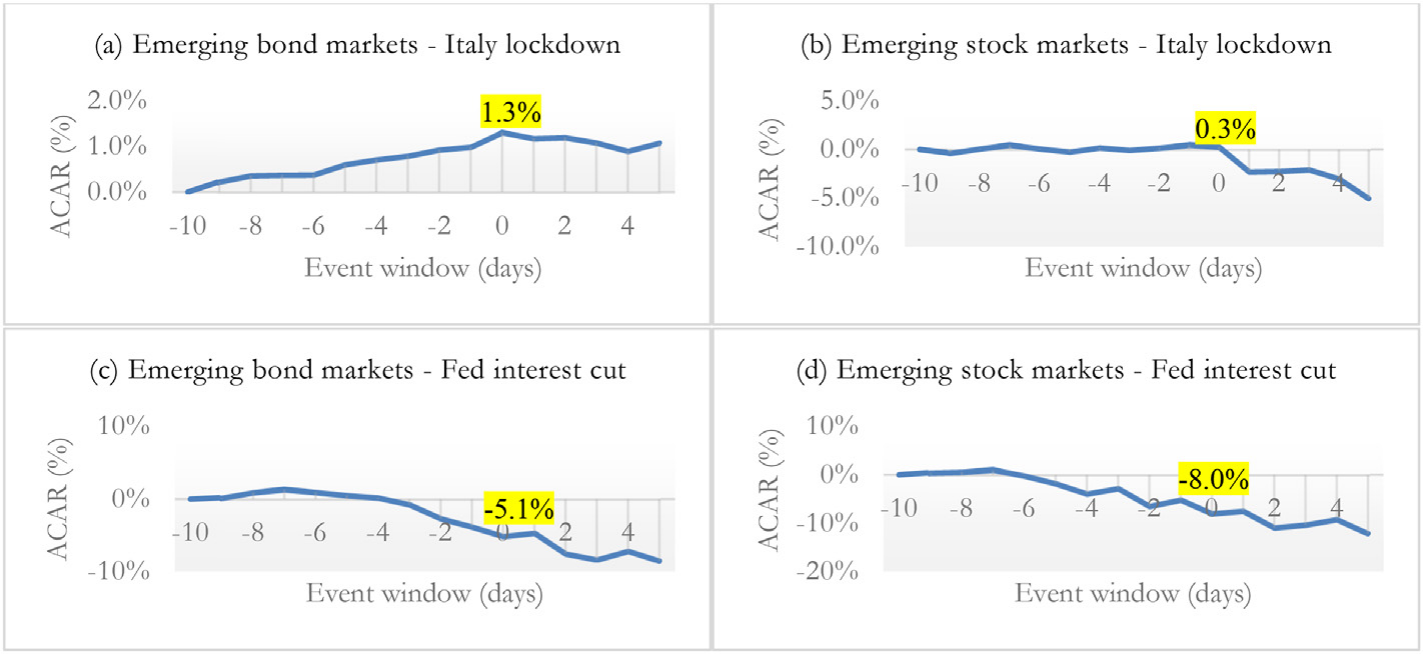
Average cumulative abnormal returns (bond yields) for emerging markets. The figure shows plots of average cumulative abnormal returns in emerging equity and bond markets following announcements by the Fed (panels (c) and (d)) and following the first lockdown in Italy (panels (a) and (b)). We use daily data for the period January 2020 through June 2020.

We now present results of event study tests on major developments in specific markets believed to be able to exert influence in global financial markets. The results of these tests therefore provide the first glimpse of possible financial contagion. Specifically, we examine two announcements: the first lockdown in Italy (the very first in Europe) declared on 22 February 2020, and the Fed's first major monetary policy pronouncement during the covid-19 crisis on 13 March 2020. Results are presented in Figure 7 for emerging capital markets. The Italy lockdown appears to exert a significant upward pressure on bond yields (panel (a)) in the emerging markets (ACAR = 1.3%, t = 12.91) on the date of announcement, a possible panic selling by emerging bond market investors. However, the day following the announcement and thereafter, a notable improvement in yields is observed, which may possibly be explained by portfolio rebalancing as investors shift from more risky assets such as equity to safer assets such as bonds.

Abnormal returns on emerging markets equity (panel (b)) decline marginally to 0.3% (t = 0.69), which is statistically insignificant, on the day of Italy's lockdown announcement. However, abnormal returns decline by a large margin (to settle at -2.3%) on the day following the announcement date, indicating possible delay in the market reaction to the announcement, reminiscent of lower levels of efficiency in emerging markets; the declines continue in the subsequent days. Over the five days leading from Italy's lockdown announcement, emerging equity markets' abnormal return exhibits a large decline to 5.34%, possibly attributable to uncertainty-induced investors' herd behavior that may have triggered a flight of capital from the riskier equity markets possibly to bond markets where yield declines are observed during the same period. The announcement by the Fed of an interest rate cut elicits more or less similar responses as the Italy lockdown announcement with equity abnormal returns falling (initially significantly to 8%, t = 6.81); however, the fall continues on the average over the next five days (panel (c)) as bond market yields increase (panel (d)), again indicating possible portfolio rebalancing.

**Figure 8:**
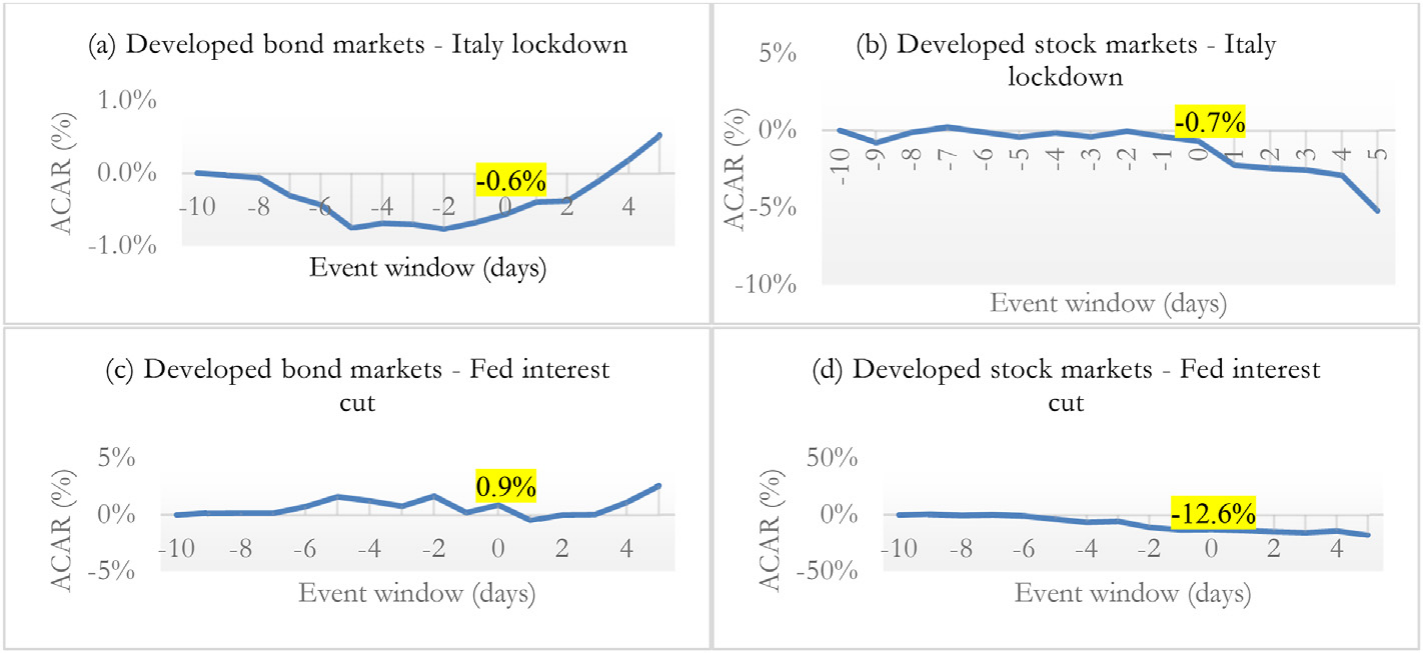
Average cumulative abnormal returns (bond yields) for developed markets. The figure shows plots of average cumulative abnormal returns in developed equity and bond markets following announcements by the Fed (panels (c) and (d)) and following the first lockdown in Italy (panels (a) and (b)). We use daily data for the period January 2020 through June 2020.

Results similar to those in emerging markets are documented for developed markets (Figure 8), where Italy lockdown announcement causes a sustained increase in bond yields (panel (a)) and a decline in equity abnormal returns (panel (b)). The Fed announcement of an interest rate cut causes high volatility in developed bond markets, the initial reaction being a sharp increment in yields (ACAR = 0.9%, t = 4.40) followed by a sharp decline that leaves ACAR in the negative territory the day after the announcement (panel (c)). However, bond yields increase subsequently, abnormal returns rising, by the fifth day, beyond those seen in the period preceding the announcement. We attribute this to panic selling as investors possibly flee to safer assets such as gold. On their part, developed equity markets reacted to the Fed interest rate cut with a sustained fall in abnormal returns, which, again, reflects possible selling pressure (panel (d)). Overall, therefore, covid-19-related announcements in major economies appear to have elicited reactions from investors both in emerging markets and in developed markets with the tendency of investors being to shift their long portfolio positions to safer assets/markets. This raises the prospects for volatility transmission across markets, a phenomenon that we examine in Section 4.3.

### 4.2 Results of regression analysis

The results presented in this section are predictive in nature because we regress the dependent variable on lagged values of independent variables. Thus, if the relationship between the dependent variable and the specific independent variable is statistically significant, we infer that known values of the regressors inform future values of the dependent variable. We control for endogeneity arising from possible omitted variables problem, using the generalized method of moments (GMM) estimation.^9^ Standard errors are adjusted for heteroskedasticity and autocorrelation using the Newey-West method, with the kernel lags set equal 4, taking into account the lag structure of the dataset. We report the outputs of three diagnostic tests: Root Mean Square Error (RMSE), Adjusted R-squared, and the J-statistic. A large RMSE, like a low R-squared, often implies low ability of the model to explain variations in the average value of the dependent variable. The Adjusted R-squared is preferred because it addresses the effect of noisy regressors that inflate the coefficient of determination without adding substantive new information. The J-statistic is from Hansen's test of overidentifying restrictions: in general, a low J-statistic indicates suitability of instrument variables.

Table 8 reports results of the model with stock returns as the dependent variable. In general, the number of covid-19 infections is not an important factor explaining stock returns in the sampled countries after controlling for other relevant variables. However, Brazil, India, New Zealand, Russia, South Africa, and USA have positive and statistically significant (at 5% and 10%) covid infection coefficients, indicating that covid-19 infections exert a mild influence on their stock market returns. This result contradicts research done earlier (Ashraf, 2020) which finds that infections were negatively related to stock returns. Our result is intuitively appealing: after the initial decline in stock returns resulting from the initial covid-19 shock (see Figure 1), the stock markets recovered quickly so that increasing returns were associated with growing number of infections and deaths in most countries. A plausible reason for the differing findings is that Ashraf's (2020) research period ends mid-April 2020 which, as observed in Figure 1, was the beginning of the stock markets' recovery while our study includes a good part of the post-recovery period.

**Table 8:** GMM results for stock returns (covid-19 form: Infections).

Country	*Const.*	$Covid_{inf}$	$X_{qs}$	$X_{ls}$	$X_{br}$	$X_{ex}$	$X_{wti}$	*RMSE*	*Adj-R*^*2*^	*J-Stat*
Brazil	0.39 (0.67)	0.01∗∗ (1.94)	0.49∗ (1.72)	0.10 (0.45)	0.04 (0.18)	0.08 (1.15)	-0.12∗∗ (-2.13)	1.50	0.05	1.51 [0.22]
China	1.46 (1.12)	0.004 (1.00)	0.01 (0.08)	-	-0.33 (-1.13)	0.17 (1.21)	0.06 (0.69)	1.06	0.02	1.93 [0.16]
India	0.67 (1.05)	0.11∗ (1.96)	0.14 (1.09)	0.10 (0.54)	0.35 (1.39)	-0.09 (-0.60)	0.13 (1.21)	1.39	0.03	0.10 [0.75]
Italy	-0.88 (-1.35)	-0,002 (-0.33)	-0.39 (-1.46)	0.07 (0.32)	0.41 (1.21)	0.04 (0.36)	0.08 (0.76)	1.43	-0.02	0.96 [0.33]
N. Z'land	0.32 (0.28)	0,09∗ (1.77)	0,12 (0.64)	-0.10 (-0.28)	-0.43 (-1.64)	0.01 (0.05)	0.09 (0.69)	1.47	0.07	2.96 [0.09]
Russia	-1.72 (-1.39)	0,01∗∗ (2.31)	-0.45∗∗ (-2.04)	-0.17 (-0.94)	0.68∗∗ (2.53)	-0.06 (-0.55)	0.15 (1.09)	1.37	0.05	0.003 [0.96]
Singapore	-0.27 (-0.32)	0,004 (1.35)	-0.01 (-0.04)	-0.04 (-1.61)	0.10 (0.44)	0.29∗ (1.75)	0.22 (1.43)	1.38	0.05	2.91 [0.09]
S. Africa	-1.24∗ (-1.88)	0,01∗∗ (2.16)	-0.19 (-0.39)	0.37 (1.61)	0.58 (1.44)	0.07 (0.61)	0.16 (1.39)	1.43	0.10	0.77 [0.38]
S. Korea	-0.01 (-0.01)	0.06 (0.83)	0.07 (0.39)	0.34 (1.45)	0.29 (1.00)	0.06 (0.50)	0.31∗ (1.76)	1.30	0.08	3.78 [0.05]
Sweden	0,19 (0.26)	-0.001 (-0.59)	0.05 (0.29)	-0.05 (-0.09)	0.34 (0.83)	-0.13 (-1.01)	-0.05 (-0.67)	1.40	-0.00	1.17 [0.28]
UK	-1,51∗ (-1.97)	0.01 (1.32)	-0.19∗ (-1.81)	0.04 (0.28)	-0.02 (-0.10)	0.14 (1.15)	0.06 (0.46)	1.42	0.00	1.10 [0.29]
USA	-2.12 (-1.31)	0,01∗ (1.82)	-0.36 (-1.55)	-0.30 (-0.96)	0.51 (1.32)	-0.24∗ (-1.97)	-0.02 (-0.27)	1.36	0.06	1.13 [0.29]

This Table reports GMM estimation results with stock returns data covering the period 01.07.2019 through 30.06.2020. The reported values are the coefficient estimates (corresponding t-statistic in parentheses); standard errors are robust to heteroscedasticity and autocorrelation. ∗, ∗∗, and ∗∗∗ indicate that the reported coefficients are statistically significant at 10%, 5% and 1% levels, respectively. The J-statistic (p-value in square braces) is from Hansen's (1982) test of overidentifying restrictions. The dependent variables are the daily stock returns of the respective countries' main stock exchange. The explanatory variables include the lagged values of: $Covid_{inf}$ representing the per million covid-19 infections; $X_{qs}$ is the quality spread, defined as the spread between ten-year treasury bonds and AAA-rated corporate bonds; $X_{ls}$ is the liquidity spread, defined as the spread between yields on ten-year Treasury bonds and three-month Treasury bills; $X_{br}$ is daily bond yields; $X_{ex}$ is exchange rate returns; and $X_{wti}$ represents the return on WTI crude oil. *Adj-R*^*2*^ is the adjusted coefficient of determination and *RMSE* is the root mean squared error; *Const.* is the regression intercept and N. Z'land is New Zealand.

For most of the stock markets, the J-statistic produces right tailed p-values between 0.1 and 0.9 and therefore, fail to reject the suitability of the instrument variables used. However, as is common with studies attempting to explain stock returns, the R-squared values are low for most markets, providing evidence of low predictability of stock returns. With regards to China, Treasury bill data are unavailable for the period under research, which impedes computation of the liquidity spread.

Table 9 reports results for regressions with covid-19 deaths as the proxy for the disease's outbreak. Results are qualitatively similar to those in Table 8 as only Brazil and India report statistically significant coefficient for covid deaths. However, there is notable improvement in the adjusted R-squared for some countries such as Brazil, South Korea, and USA. The J-statistic largely fails to reject the instruments. It is interesting to explore the negative and significant coefficient for WTI crude oil returns for Brazil. The literature suggests that for oil-exporting economies (such as Brazil), a rise in oil prices should have a positive effect on stock returns (Park and Ratti, 2008), predicated on the income effect: higher incomes around the world increase demand for oil which results in price increases that more than offset the negative cost effects on oil prices (Degiannakis et al., 2017). Oil prices declined sharply in the early part of 2020 due to low demand, as many countries went into lockdowns and airlines got grounded, before beginning to rise gradually towards the end of April. However, Brazilian stock prices only fell briefly between February and March 2020, the rest of the time appearing to have a negative correlation with oil prices perhaps due to the diversified nature of the country's economy.

**Table 9:** GMM results for stock returns (covid-19 form: Deaths).

Country	*Const.*	$Covid_{dth}$	$X_{qs}$	$X_{ls}$	$X_{br}$	$X_{ex}$	$X_{wti}$	*RMSE*	*Adj-R*^*2*^	*J-Stat*
Brazil	0.41 (0.92)	0.15∗∗∗ (2.69)	0.45∗ (1.82)	-0,10 (-0.54)	0.12 (0.76)	0.02 (0.45)	-0.11∗∗ (-2.16)	0.88	0.13	0.06 [0.80]
China	0.79 (1.24)	0.01 (0.28)	0.03 (0.41)	-	-0.15 (-1.26)	0.11 (1.02)	0.08 (0.96)	1.23	-0.00	2.10 [0,15]
India	0.96 (1.37)	1.10∗ (1.78)	0.21 (1.43)	0.04 (0.15)	0.02 (0.07)	-0.12 (-0.67)	0.10 (0.72)	-0.00	0.01	1.24 [0.26]
Italy	-0.84 (-1.24)	0.01 (0.15)	-0.30 (-1.19)	0.06 (0.24)	0.38 (0.90)	0.03 (0.26)	0.08 (0.82)	1.52	-0.01	1.05 [0.31]
N. Z'land	0.77 (0.49)	0.23 (0.75)	0.14 (0.57)	0.08 (0.76)	-0.17 (0.62)	-0.04 (0.73)	-0.10 (0.37)	0.79	-0.08	1.94 [0.16]
Russia	0.77 (0.35)	-0.50 (-1.52)	-0.78 (-1.52)	0.00 (-0.02)	0.86 (1.51)	0.00 (-0.03)	-0.00 (-0.00)	0.90	-0.02	0.17 [0.68]
Singapore	-0,96 (-0.85)	0.82 (0.58)	-0.06 (-0.36)	-0.02 (-0.56)	-0.25 (-0.84)	0.29 (1.53)	0.24 (1.49)	1.51	0.03	2.80 [0.09]
S. Africa	-0.73 (-0.65)	0.03 (0.07)	-0.70 (0.42)	0.04 (0.89)	0.78 (1.00)	0.11 (1.53)	0.06 (0.50)	0.91	-0.04	0.83 (0.36)
S. Korea	1.03 (1.32)	-2.65 (-0.68)	0.18 (0.95)	0.06 (0.36)	0.58 (1.63)	0.10 (0.70)	0.24 (1.37)	1.39	0.10	2.17 [0.14]
Sweden	-0.41 (-0.46)	0.01 (0.32)	-0.07 (-0.40)	-0.67 (-1.19)	-0.03 (-0.07)	-0.15 (-1.10)	-0.11 (-1.44)	1.28	-0.04	1.29 [0.26]
UK	-2.41 (-1.29)	0.04 (0.82)	-0.27 (-1.42)	0.18 (1.08)	-0.31 (-0.85)	0.20 (1.47)	0.02 (0.14)	1.45	-0.01	0.98 [0.32]
USA	-0.10 (-0.05)	0.07 (1.17)	-0.46∗∗ (-2.02)	-0.25 (-0.41)	-1.02 (-1.32)	-0.25∗∗ (-2.15)	-0.03 (-0.38)	1.47	0.10	1.28 [0.25]

This Table reports GMM estimation results with stock returns data covering the period 01.07.2019 through 30.06.2020. The reported values are the coefficient estimates (corresponding t-statistic in parentheses); standard errors are robust to heteroscedasticity and autocorrelation. ∗, ∗∗, and ∗∗∗ indicate that the reported coefficients are statistically significant at 10%, 5% and 1% levels, respectively. The J-statistic (p-value in square braces) is from Hansen's (1982) test of overidentifying restrictions. The dependent variables are the daily stock returns of the respective countries' main stock exchange. The explanatory variables include the lagged values of: $Covid_{dth}$ representing the per million covid-19 deaths; $X_{qs}$ is the quality spread, defined as the spread between ten-year treasury bonds and AAA-rated corporate bonds; $X_{ls}$ is the liquidity spread, defined as the spread between yields on ten-year Treasury bonds and three-month Treasury bills; $X_{br}$ is daily bond yields; $X_{ex}$ is exchange rate returns; and $X_{wti}$ represents the return on WTI crude oil. *Adj-R*^*2*^ is the adjusted coefficient of determination and *RMSE* is the root mean squared error; *Const.* is the regression intercept and N. Z'land represents New Zealand.

In Table 10, we use a dummy variable taking a value of 1 after the first infection and 0 before, to represent the covid-19 outbreak in each country. The results affirm the null hypothesis that covid-19 did not impact stock returns. The J-statistic does not reject any specification and all R-squared values are below 10%. Of the remaining variables, oil returns affect stock returns negatively in Brazil and positively in South Africa. The positive effect in South Africa may be attributed to the lower crude oil prices which may have been favorable to the then struggling economy, possibly aiding recovery. Currency returns have a positive impact on stocks in China and a negative impact for USA stocks. Interestingly, quality spreads negatively affect stock returns in Russia and bond returns negatively affect stock returns in China, the latter result being consistent with the hypothesis of flight from the stocks to the relatively safer bond markets.

**Table 10:** GMM results for stock returns (covid-19 form: dummy variable).

Country	*Const.*	$Covid_{dum}$	$X_{qs}$	$X_{ls}$	$X_{br}$	$X_{ex}$	$X_{wti}$	*RMSE*	*Adj-R*^*2*^	*J-Stat*
Brazil	0.87 (1.46)	-0.48 (-1.31)	0.27 (1.29)	0.05 (0.43)	0.02 (0.12)	0.04 (0.75)	-0.10∗ (-1.88)	0.97	0.03	1.12 [0.29]
China	1.69∗∗ (2.54)	-0.47∗∗ (-2.08)	0.08 (1.15)		-0.25∗∗∗ (-2.61)	0.16∗∗ (2.56)	0.06 (0.84)	0.97	0.03	1.86 [0.17]
India	0.85 (1.39)	-0.17 (-0.84)	0.15 (1.47)	0.01 (0.16)	-0.05 (-0.41)	-0.08 (-0.89)	0.11 (1.07)	0.97	0.02	0.03 [0.85]
Italy	-0.33 (-0.63)	-0.04 (-0.18)	-0.13 (-0.81)	-0.15 (-1.06)	0.27 (1.28)	0.01 (0.08)	0.08 (0.83)	0.98	-0.002	0.75 [0.38]
N. Z'land	0.28 (0.29)	0.30 (0.51)	0.06 (0.41)	-0.14 (-0.74)	-0.03 (-0.16)	0.08 (1.05)	0.12 (0.95)	0.98	0.01	3.29 [0.07]
Russia	0.90 (0.88)	0.09 (0.46)	-0.33∗ (-1.74)	-0.09 (-0.82)	0.30 (1.36)	-0.08 (-0.78)	0.16 (1.19)	0.97	0.03	0.44 [0.51]
Singapore	0.40 (0.51)	-0.39 (-1.56)	0.04 (0.35)	-0.03 (-1.19)	-0.14 (-0.86)	0.19 (1.72)	0.20 (1.43)	0.95	0.07	3.54 [0.06]
S. Africa	-0.05 (-0.12)	-0.36 (-1.18)	-0.13 (-0.41)	0.02 (0.12)	0.40 (1.31)	0.09 (1.65)	0.17∗ (1.72)	0.96	0.05	0.12 [0.72]
S. Korea	0.13 (0.15)	-0.19 (-0.80)	0.01 (0.04)	0.11 (0.86)	-0.05 (-0.75)	0.10 (1.35)	0.22 (1.28)	0.95	0.07	3.68 [0.06]
Sweden	0.09 (0.20)	0.06 (0.32)	0.02 (0.24)	0.10 (1.14)	-0.04 (-0.47)	-0.04 (-0.43)	-0.01 (-0.17)	0.99	-0.02	0.56 [0.45]
UK	-0.78 (-1.18)	-0.11 (-0.44)	-0.12 (-1.32)	0.11 (1.23)	-0.03 (-0.36)	0.13 (1.61)	0.06 (0.63)	0.98	0.01	0.88 [0.34]
USA	-0.05 (-0.05)	-0.29 (-1.51)	-0.11 (-0.71)	0.00 (0.03)	-0.08 (-0.50)	-0.15∗ (-1.79)	-0.03 (-0.47)	0.97	0.03	1.04 [0.31]

This Table reports GMM estimation results with stock returns data covering the period 01.07.2019 through 30.06.2020. The reported values are the coefficient estimates (corresponding t-statistic in parentheses); standard errors are robust to heteroscedasticity and autocorrelation. ∗, ∗∗, and ∗∗∗ indicate that the reported coefficients are statistically significant at 10%, 5% and 1% levels, respectively. The J-statistic (p-value in square braces) is from Hansen's (1982) test of overidentifying restrictions. The dependent variables are the daily stock returns of the respective countries' main stock exchange. The explanatory variables include the lagged values of: $Covid_{dum}$ is a dummy variable that takes the value of zero before the first infection and 1 thereafter; $X_{qs}$ is the quality spread, defined as the difference between ten-year treasury bonds and AAA-rated corporate bonds; $X_{ls}$ is the liquidity spread, defined as the difference between yields on ten-year Treasury bonds and three-month Treasury bills; $X_{br}$ is daily bond yields; $X_{ex}$ is exchange rate returns; and $X_{wti}$ represents the return on WTI crude oil. *Adj-R*^*2*^ is the adjusted coefficient of determination and *RMSE* is the root mean squared error; *Const.* is the regression intercept and N. Z'land represents New Zealand.

Given the finding on the relationship between bond returns/quality spreads and stock returns in some emerging markets, it is interesting to ascertain the role of the covid-19 outbreak on bond yields. Portfolio theory postulates a positive relationship between asset returns and risk. In the bond markets, the risk of default and possibly sovereign risk in some markets may have heightened with the economic lockdowns and associated with covid-19 which had perverse effects on economic output. Thus, the *a priori* expectation is that of a positive relationship between covid (especially infections) and bond yields particularly in emerging and small developed markets, where portfolio revisions may have been, at least in the initial stages, in the form of short positions. Table 11 reports the abridged results of the relationship between bond yields and covid-19 infections and deaths and a dummy variable.

**Table 11:** GMM results for bond yields.

Country	(a): covid-19 infections			(b): covid-19 deaths			(c): covid-19 dummy			Control variables and intercept
	$Covid_{inf}$	*Adj-R*^*2*^	*J-Stat*	$Covid_{dth}$	*Adj-R*^*2*^	*J-Stat*	$Covid_{dum}$	*Adj-R*^*2*^	*J-Stat*	
Brazil	-0.01∗∗ (-2.47)	0.52	0.02 [0.89]	-0.12 (-1.61)	0.55	1.30 [0.25]	-0.52∗∗ (-2.19)	0.62	0.004 [0.95]	Yes
China	0.01∗∗∗ (6.91)	0.52	0.86 [0.35]	-0.04 (-0.65)	0.05	0.25 [0.62]	-1.82∗∗∗ (-13.92)	0.78	0.08 [0.77]	Yes
India	-0.19∗∗∗ (-5.72)	0.65	0.27 [0.61]	-2.49∗∗ (-2.26)	0.31	0.25 [0.62]	-1.55∗∗∗ (-8.84)	0.65	0.02 [0.88]	Yes
Italy	0.02∗∗∗ (5.82)	0.82	0.01 [0.93]	0.08∗∗∗ (4.12)	0.66	0.04 [0.84]	0.28 (1.00)	0.79	0.12 [0,72]	Yes
N. Z'land	0.06∗∗∗ (3.78)	0.25	0.25 [0.62]	-0.49∗ (-1.75)	0.63	0.58 [0.45]	-2.04∗∗∗ (-10.93)	0.65	0.02 [0.89]	Yes
Russia	-0.01∗∗∗ (-4.67)	0.80	0.53 [0.47]	-0.17 (-1.34)	0.92	0.43 [0.51]	-0.72∗∗∗ (-7.32)	0.83	0.20 [0.65]	Yes
Singapore	-0.01∗∗∗ (-4.07)	0.55	0.11 [0.74]	-1.63∗∗ (-2.34)	0.11	0.01 [0.91]	-1.42∗∗∗ (-7.92)	0.71	0.00 [0.96]	Yes
S. Africa	-0.01∗∗ (-2.41)	0.75	0.64 [0.42]	-0.08 (-1.32)	0.94	0.06 [0.80]	-0.11 (-0.85)	0.89	0.27 [0.60]	Yes
S. Korea	-0.16∗∗∗ (-5.32)	0.35	2.84 [0.09]	6.21∗∗∗ (4.38)	0.37	0.03 [0.85]	-1.40∗∗∗ (-5.12)	0.33	0.02 [0.88]	Yes
Sweden	0.02∗∗ (2.52)	0.74	2.41 [0.12]	-0.01 (-1.66)	0.32	1.76 [0.18]	0.85∗∗∗ (3.35)	0.73	0.00 [0.97]	Yes
UK	-0.01 (-1.40)	0.24	0.86 [0.35]	0.03∗∗∗ (2.97)	0.44	0.01 [0.93]	-1.73∗∗∗ (-7.41)	0.60	0.02 [0.88]	Yes
USA	-0.01∗∗∗ (-8.00)	0.76	0.03 [0.95]	-0.03∗∗∗ (-3.46)	0.29	2.49 [0.11]	-1.08∗∗∗ (-5.12)	0.83	0.01 [0.93]	Yes

This Table reports GMM estimation results with bond yield data covering the period 01.07.2019 through 30.06.2020. The reported values are the coefficient estimates (corresponding t-statistic in parentheses); standard errors are robust to heteroscedasticity and autocorrelation. ∗, ∗∗, and ∗∗∗ indicate that the reported coefficients are statistically significant at 10%, 5% and 1% levels, respectively. The J-statistic (p-value in square braces) is from Hansen's (1982) test of overidentifying restrictions. The dependent variables are the daily stock returns of the respective countries' main stock exchange. The explanatory variables include values of: $Covid_{dth}$ representing the per million covid-19 deaths; $Covid_{inf}$, representing the per million covid-19 infections; and $Covid_{dum}$, a dummy variable that takes a value of zero before the first infection and 1 after, respectively and lagged values of $\;X_{qs}$, the quality spread, defined as the difference between ten-year treasury bonds and AAA-rated corporate bonds; $X_{ls}$, the liquidity spread, defined as the difference between yields on ten-year Treasury bonds and three-month Treasury bills; $X_{br}$, daily bond yields; $X_{ex}$, exchange rate returns; and $X_{wti}$, the return on WTI crude oil. *Adj-R*^*2*^ is the adjusted coefficient of determination; *RMSE* is the root mean squared error; *Const.* is the intercept; N. Z'land represents New Zealand.

On the average, bond yields were falling in developed markets during the period of covid (January through June 2020), except for a few markets such as Italy, and were largely stable in emerging markets (Figure 1). Thus, the relationship between bond yields and covid-19 presence (dummy), deaths, and infections is expected to vary by country. The effect of covid infections, denoted $Covid19_{inf}$, is significant in many regressions (panel (a)), many of them negative, consistent with the pattern observed in Figure 1. That is, once the initial shock of covid-19 was registered (see event studies results) in the bond markets, bond yields continued on their long-term declining trend in many countries regardless of further announcements of new infections. Thus, although the number of covid-19 infections were growing during the period, the growth was associated with falling bond yields, giving the negative relationships. This finding is consistent with Haddad et al. (2020), who report that covid-19 disruptions of the US market were transient and disappeared as soon as monetary policy authorities intervened.

A similar inference follows for covid-19 deaths (panel (b)). However, in countries that experienced a covid-19 health crisis during the period, such as the UK and Italy, the number of deaths were expectedly associated with yield increases because lockdown measures instituted in those countries to manage the health crises precipitated investor fears about economic declines. In panel (c), where covid-19 is represented by a dummy variable, it is also evident that a negative relationship dominated; however, probably because of its then unorthodox ways of managing the disease, Sweden witnesses a significant positive relationship, which may be attributed to investor concern about effect of the relaxed covid health policy and a concomitant possible higher infection rates on near-term economic productivity.

### 4.3 Results of contagion tests

#### 4.3.1 Return spillovers

We start by discussing estimation results for Eq. (3).^10^ Focusing initially on equity returns, India, South Africa, and USA are the only countries with diagonal values that are statistically significant. Thus, of the sampled markets, only their stock returns depend on their own history suggesting the existence of their own spillovers during the sample period. All the mean return spillovers are negative and high, indicating a reversal of fortunes for these markets. For the remaining countries for which own spillovers are insignificant, the conditional volatility of returns is “imported” from other markets (Theodossiou and Lee, 1993). Thus, our next discussion explores cross-mean spillovers to establish whether they may have emanated from our sampled markets.

Significant cross-mean spillovers are reported for all countries. Spillovers from the Indian stock market are evident in all sampled markets. India was arguably the worst affected economy by the covid-19 outbreak and its stock witnessed one of the worst volatilities during 2020 with, for example, the Sensex index dropping by 13.2% and the Nifty index falling by 29% on March 23, 2020 (Bora and Basistha, 2021). The Indian stock market is, however, most significantly affected by USA stock returns (coefficient = 0.2954; standard error = 0.1009). The results further show that South Africa's stock return spillovers are mainly transmitted by other developing countries except China; this seems to be the case for Brazil as well, with the exception of mean spillovers from Singapore (at the 1% level) and Italy (at 10%).

We pay a close attention to Italy because of its earlier-discussed lockdown announcement. The results show that Italy was a source of return spillovers to Brazil at the 10% significance level and to India at 1%. We are also interested to know whether USA, because of its traditional role as a source of global volatility (Vo and Tran, 2020) and given our earlier interest in its announcement of interest rates cut, presents spillover effects to other markets. We document mean return spillovers from the USA to six of the sampled markets, both developed (Singapore, New Zealand, and Sweden) and developing (India, China, South Korea).

Turning next to bond yields, Russia, India, and Sweden have statistically significant diagonal entries. The own mean spillover effect is negative and high for India and Sweden which indicates a downward drift in these bond markets. With regards to cross mean yield spillovers, we find, expectedly, that the USA bond market is the most dominant source of yield transmission. Aside from Sweden and South Africa, the off-diagonal parameters representing cross-mean spillovers from USA to the other markets, are statistically significant, consistent with the findings of previous research (e.g., Hakim and McAleer, 2010). However, majority of the off-diagonal parameters are insignificant for developing markets.

Of developed markets, New Zealand's yields are the most affected by spillovers with Brazil, Russia, and South Africa transmitting the most significant effects. In studying the relationship between major bond markets, Hunter and Simon (2005) find that while mean and volatility spillovers were evident between major international bond markets, they are weaker than those evidenced in stock markets: consistently, our results show that Italy and USA exert the most pressure on other developed markets, but overall, cross mean spillovers in developed countries seem much weaker for bonds than the effect in the stock markets during the covid-19 crisis.

### 4.4 Volatility spillovers

The multivariate GARCH-BEKK model, $H_{t}=C+A^{\prime }\left(\varepsilon_{t-1}\varepsilon_{t-1}^{\prime }\right)A+B^{\prime }H_{t-1}B$, enables us to determine the direction of transmission of volatility. Diagonal values in the matrix A indicate own volatility effects (ARCH) and the diagonal values in the matrix B (GARCH effects) indicate persistence in own conditional volatility.^11^ With the stock markets, all the diagonal vectors, except for India, Sweden, and USA, are statistically significant. This indicates that the presence of stock returns volatility spillovers responds significantly to their own volatility from previous periods. Further, with regards to the presence of GARCH effects, that is, the persistence of volatility, all the markets, except China, exhibit dependence on their own volatility in the previous period.

The off-diagonal coefficients of A and B capture cross-market effects of volatility spillovers. USA exhibits high unidirectional linkages in the transmission of shocks to all the markets except South Korea, which is more significantly affected by other emerging markets. The USA exports as high as 33% (to Russia) of its volatility. This result is not surprising as the S&P 500 has been found to be the major transmitter of volatility worldwide (see, e.g., Ling and Gurjeet, 2010). Over the last two decades, there has been substantial financial integration, with USA being central to it (Li and Giles, 2014). As a result, during the covid-19 period, volatility transmission has been significant as international portfolio investors revise their positions in various markets. The fact that no bidirectional volatility is noted for USA indicates that it was the source of the financial contagion that affected many stock markets during the sampled period.

**Figure 9:**
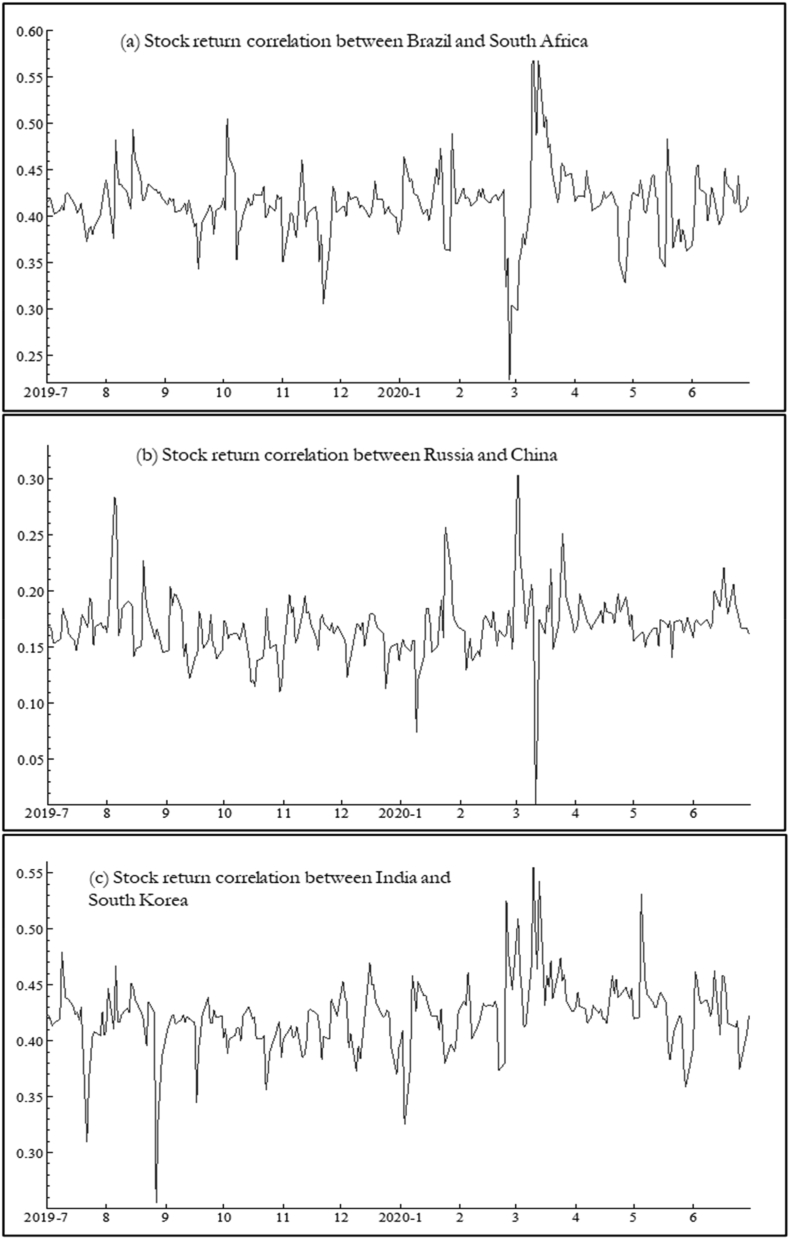
Dynamic conditional correlations between stock returns in pairs of developing markets. This figure reports results of conditional correlations between returns on developing stock markets of Brazil and South Africa (panel (a)), Russia and China (panel (b)) and India and South Korea (panel (c)) using daily returns for the period July 2019 through June 2020.

The GARCH effects, however, are not as significant: only 7 countries show significant (1% and 5% levels) off-diagonal entries in relation to the cross-market volatility spillovers. Russia, India, and China, notably, are among the countries with no significant volatility persistence. When analysing conditional correlations, financial contagion is detected through significant increase of stock price co-movements. Figure 9 highlights the conditional correlation between selected emerging equity market pairs, namely Brazil and South Africa (panel (a)), Russia and China (panel (b)) and India and South Korea (panel (c)). Although volatility increases are visible during the initial period of covid-19 outbreak (early 2020), the increment in volatility does not seem to persist after the initial shock. However, even pre-crisis, there is notable correlation among the emerging market countries (above 20% in the three highlighted pairs), the disease outbreak shock only serving to increase the correlation although, in many cases, for a brief period. In all three cases, episodes of volatility clustering are also evident which seem to coincide with release of major news in the country-pairs.

**Figure 10:**
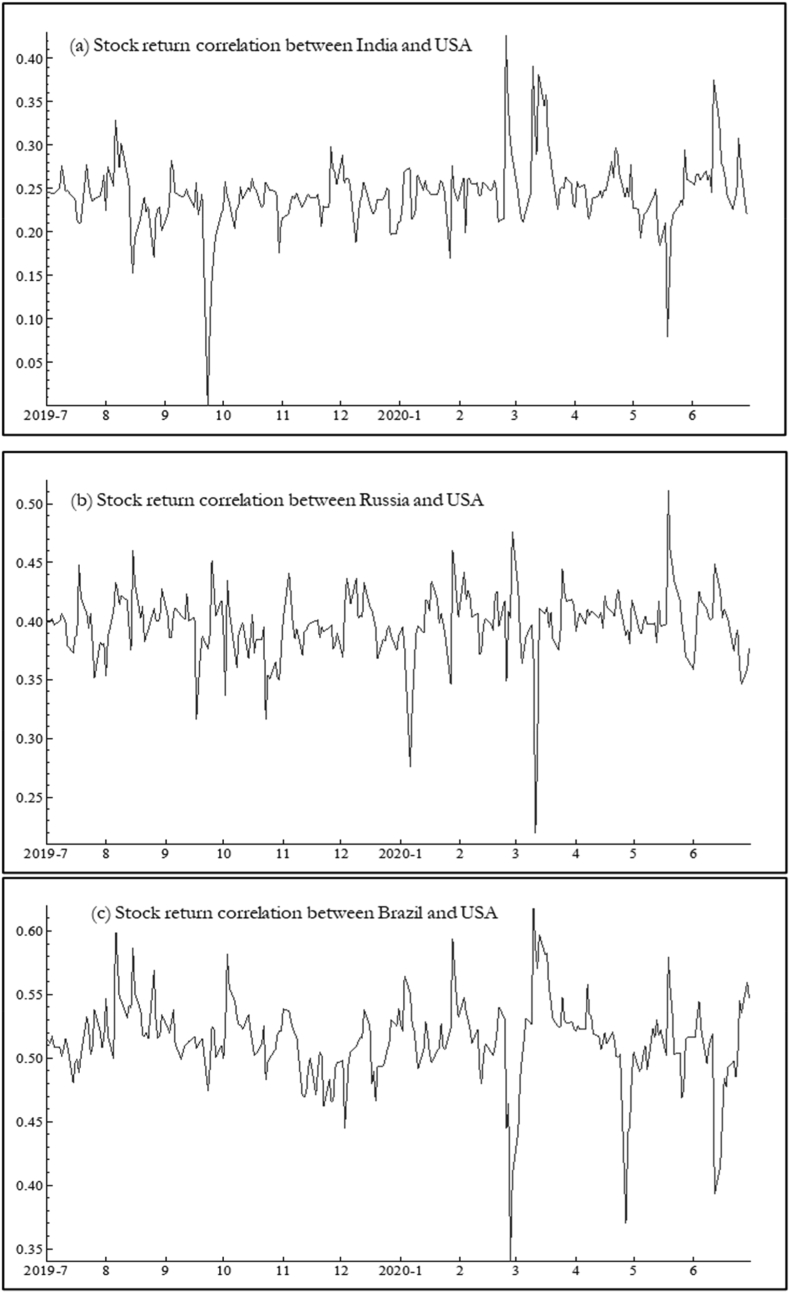
Dynamic conditional correlations between stock returns in developing markets and the USA. This figure reports results of conditional correlations between returns on developing and advanced stock markets of India and USA (panel (a)), Russia and USA (panel (b)) and Brazil and USA (panel (c)) using daily returns for the period July 2019 through June 2020.

Figure 10 describes correlations between USA and selected emerging market returns: India (panel (a)), Russia (panel (b)) and Brazil (panel (c)). We find strong evidence of correlation between USA and the BRICS markets during the covid-19 outbreak. Zhang, Li and Yu (2013) found that, contrary to India and China, Russia and Brazil's stock markets had stronger correlations with developed countries. Consistently, our results show that correlations between Russia and Brazil with USA are generally larger than the correlation between USA and India. We observe a sharp negative correlation between Russia and USA markets in mid-March 2020, when Russia's first covid-19 wave essentially began^12^ and between Brazil and USA towards the end of February 2020 when the first case of the disease was reported in Brazil.^13^ Negative correlation during periods of uncertainty signify the presence of “flight to quality” rather than contagion (Baur and Lucey, 2009). This therefore suggests that at the onset of the disease, investors retreated significantly from these emerging markets. Spikes in positive correlation are evident in the USA-India series during the month of March 2020 which possibly indicate volatility transmission between the two markets. Given our findings, it is reasonable to infer that the source of the contagion is the USA market.

**Figure 11:**
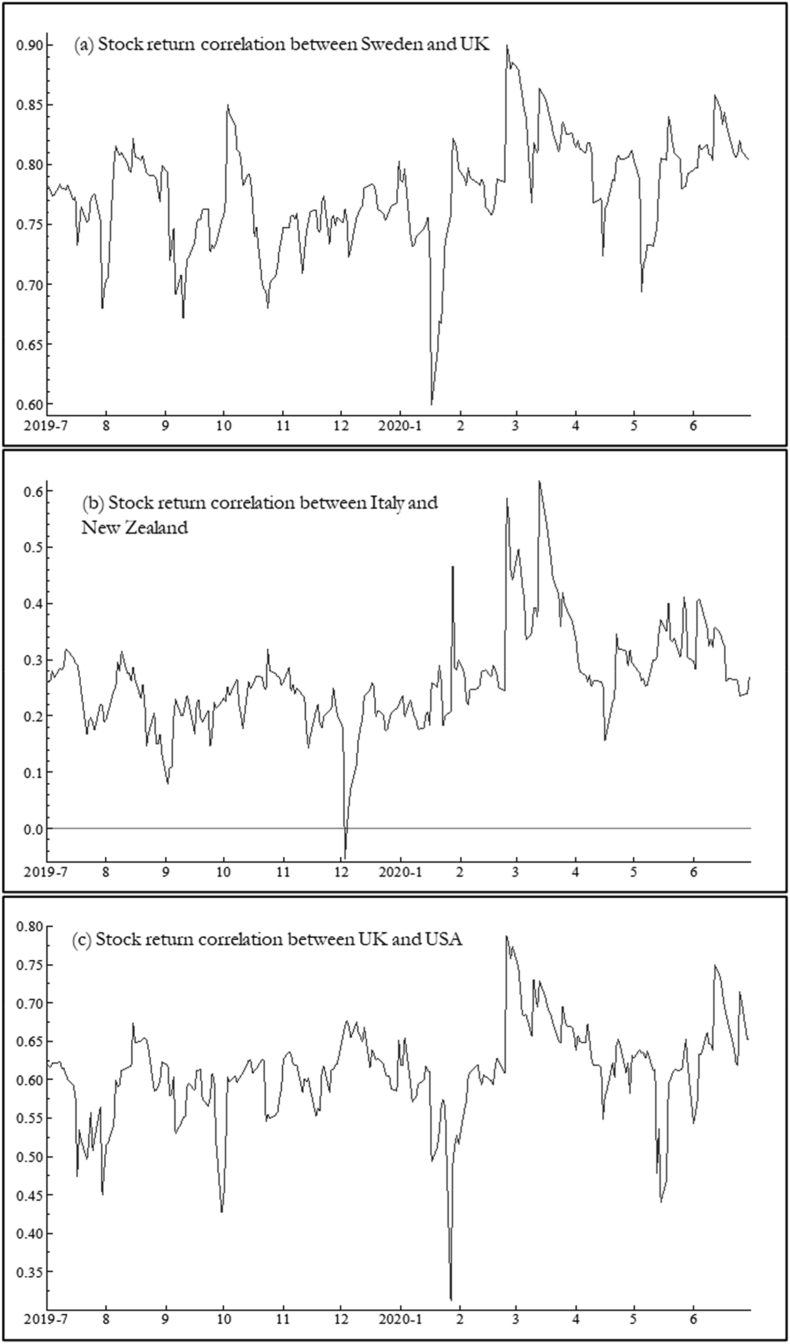
Dynamic conditional correlations between stock returns in developed markets. This figure reports results of conditional correlations between returns on advanced stock markets of Sweden and the UK (panel (a)), Italy and New Zealand (panel (b)) and UK and USA (panel (c)) using daily returns for the period July 2019 through June 2020.

Turning next to the estimation outputs for bond yields, the diagonal parameters are significant for Russia, South Africa, Italy, Singapore, Sweden, and USA. Own spillovers range from -6% (South Africa) to 51% (Singapore). For these countries, the volatility of bond yields is highly dependent on their own volatility in the preceding period. The diagonal B entries are statistically significant, therefore highlighting the presence of own volatility persistence. There are many significant off-diagonal coefficients, which indicate the presence of a high degree of volatility spillovers across markets. That is, the linkages are significantly bidirectional when it comes to the transmission of shocks. This finding supports existing evidence of the integration of bond markets and that no single market is at the “centre” of financial linkages (Gilmore et al., 2010). Thus, although spillover effects are evident, with no single direction of volatility being ascertained, the hypothesis of the presence of financial contagion between developed and emerging bond markets during this period appears unsupported. In contrast, high correlations, which appear to increase during the covid-19 period, are reported between developed equity markets (Figure 11), such as, for example, Sweden and the UK (panel (a)), Italy and New Zealand (panel (b)) and UK and USA (panel (c)), indicating possible contagion effects between developed equity markets.

**Figure 12:**
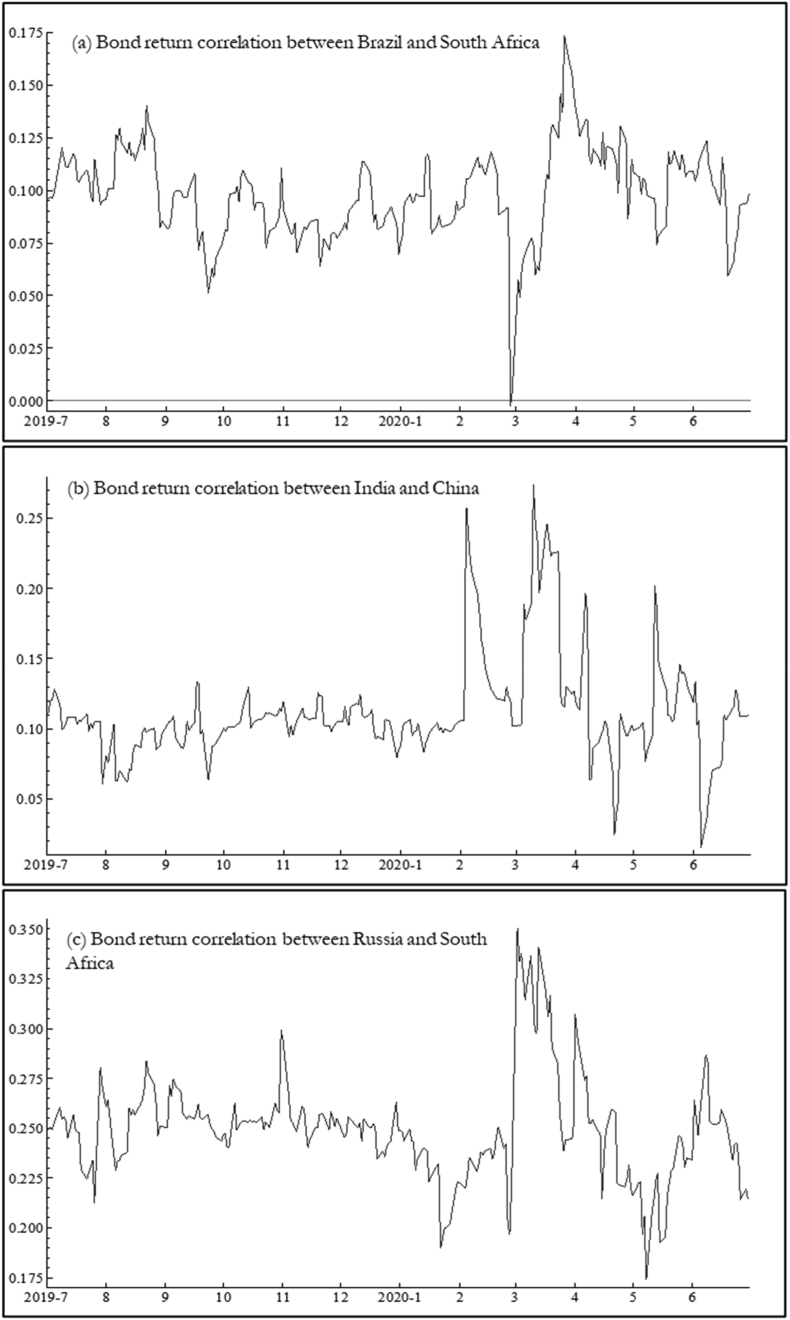
Conditional correlation between bond yields in selected emerging markets. This figure reports results of conditional correlations between yields on developing bond markets of Brazil and South Africa (panel (a)), India and China (panel (b)) and Russia and South Africa (panel (c)) using daily yields for the period July 2019 through June 2020.

Exploring conditional correlations in bond yields across pairs of markets, we start, as in the case of equity markets, with the within-emerging markets analysis. Figure 12 reports the results for three pairs of emerging markets, namely, Brazil and South Africa (panel (a)), India and China (panel (b)) and Russia and South Africa (panel (c)). The figure shows that, on average, correlations between the markets increased noticeably between February 2020 to around the end of April 2020. The increased volatility appears to coincide with the commencement of the covid-19 outbreak in many of the sampled countries suggesting that the disease may have induced volatility transmission across these markets.

**Figure 13:**
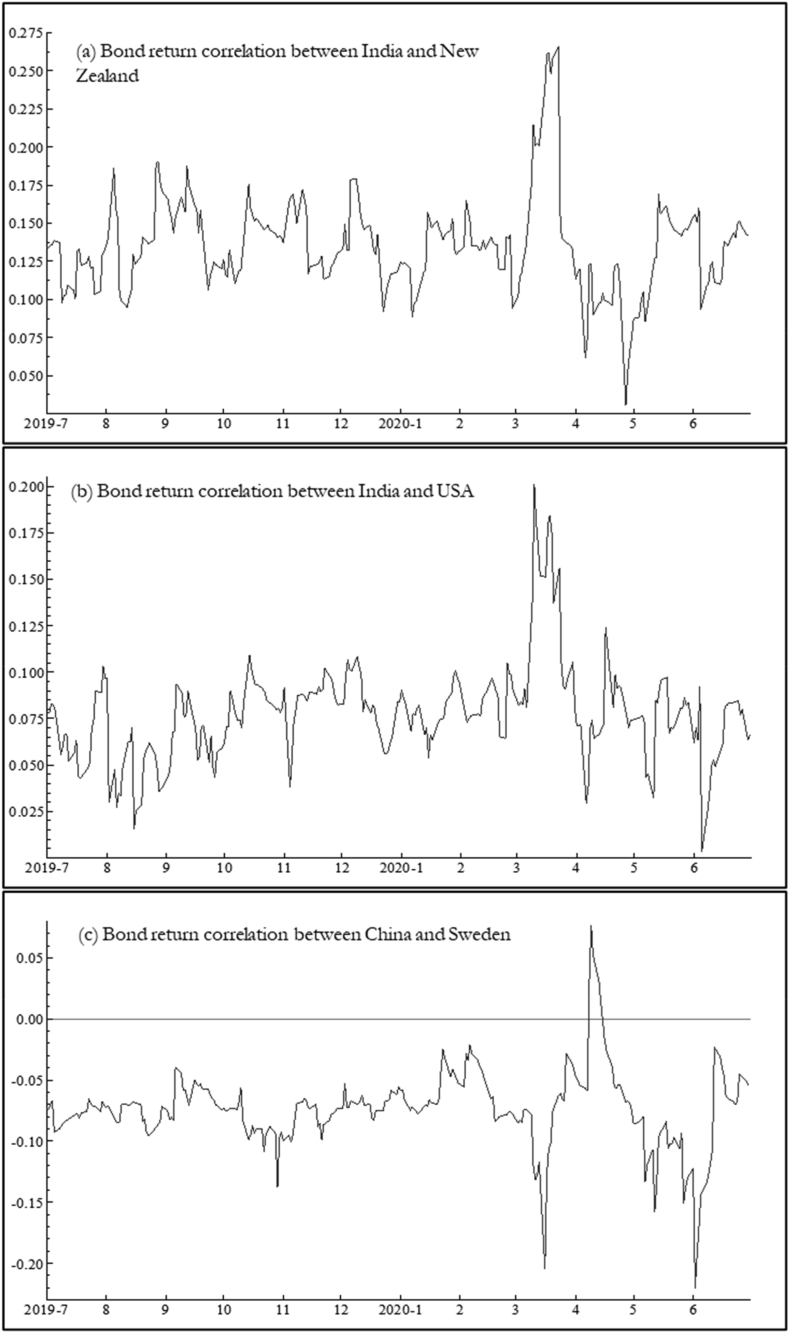
Conditional correlation between bond yields in selected emerging and developed markets. This figure reports results of conditional correlations between yields on developing and advanced bond markets for, respectively, India and USA (panel (a)), India and New Zealand (panel (b)) and China and Sweden (panel (c)). The data are for the period July 2019 through June 2020.

**Figure 14:**
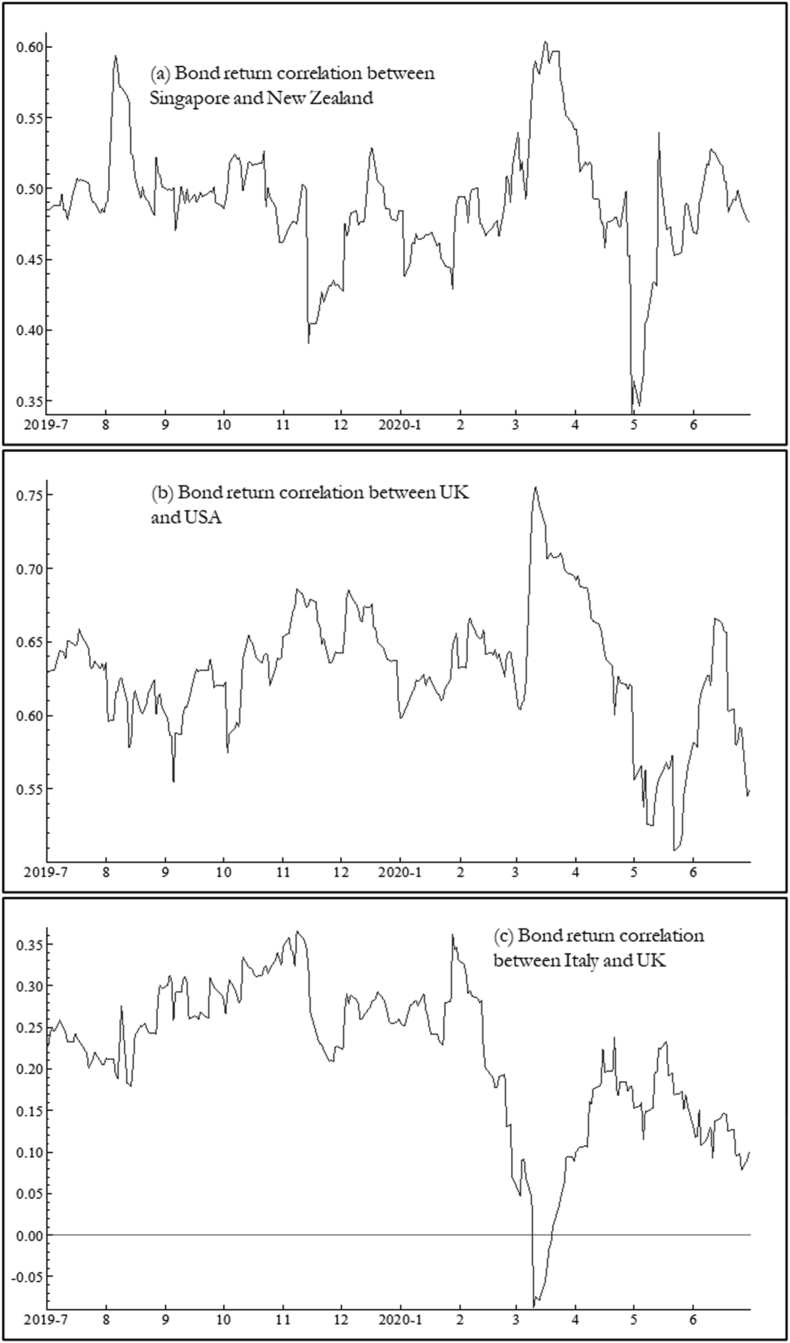
Conditional correlation between bond yields in advanced markets. This figure reports results of conditional correlations between yields on developing and advanced bond markets for, respectively, Singapore and New Zealand (panel (a)), the UK and USA (panel (b)) and Italy and the UK (panel (c)). The data are for the period July 2019 through June 2020.

In Figure 13 where we report the conditional correlation between bond yields in emerging and developed markets, high correlations are evident during March 2020, going to as high as 27% (in the case of India and New Zealand (panel (a)). This shows possible volatility transmission between the two sets of markets. Broadly similar results are observed between India and USA (panel (b)). However, the case of China and Sweden (panel (c)), which reports negative correlation in mid-March 2020 is interesting given the initial hesitancy of the Swedish government to use the more stringent “lockdown” measures. Happening when China had adopted one of the most restrictive lockdown measures in one of its more affected provinces, Sweden could have been one of the leading recipients of portfolio flows of international investors dumping the Chinese bond market at the time. In the case of Italy and UK (Figure 14, panel (c)), where a 40% drop in correlation is observed between February and March 2020, we believe that the low correlation could also be explained by the UK's exit from the European Union. Observations of a similar nature are made for correlations between the other developed market pairs whose results are reported in Figure 14, such as Singapore and New Zealand (panel (a)), the UK and USA (panel (b)).

## 5 Conclusions

In this paper, we study the effects of the covid-19 outbreak on the returns on capital assets, namely, bonds and stocks. We adopt a comparative analysis approach involving developed markets (Italy, New Zealand, Singapore, Sweden, USA, and UK) and emerging markets (Brazil, China, India, Russia, South Africa, and South Korea). We use daily data for a sample period from 01.07.2019 through 30.06.2020. The paper attempts to address the issues posed using different alternative methodologies such as events study, regression analysis, and the GARCH-BEKK model. The first part of the event study examines three covid-19-related events, namely, the first infection, first death, and the lockdown announcement in each country. We find that the flight-to-safety phenomenon is more manifest in emerging countries as both the first infection and lockdown negatively affect stock returns but have a positive effect on bond yields. The first event study therefore, demonstrates that covid-19 had a significant effect on asset returns following announcements regarding infections and lockdowns, with lockdowns having a much more profound effect. Our results are consistent with Aizenman et al. (2016) who find negative effects on stock returns as a result of the global financial crisis of 2017/9.

In the second event study, we analyze the effect of the first lockdown announcement in Italy (also the first in the European Union) and the effect of the Fed (USA) announcement to cut interest rates. The second announcement provides initial support for possible contagion affecting markets as anticipation of this news appear to be impounded into returns days before the announcement was made. With regards to the first lockdown announcement in Italy, however, the ACAR results for stocks in both developing and emerging markets are not statistically significant, therefore, ruling out the event as a potential source of volatility in the stock markets.

The regression analysis makes use GMM estimation. Covid-19 takes three separate forms, namely, covid infections per million, covid deaths per million and a dummy variable taking the value of 1 after the first infection in a country and zero elsewhere. We find that the independent variables used had a greater predictability power in bond yields than in stock returns. We also find that, when COVID-19 takes the form of infections, it has a significant positive effect on markets such as the Brazil, India, New Zealand, Russia South Africa, and USA. This result is interpreted as follows: once the initial shock was absorbed by the market (evident in the event study), the stock markets started recovering so that increased infections became associated with increased returns. The effect of covid-19 deaths on the stock market is contrarily much muted. However, markets such as Brazil and India see a positive relationship with their stock returns.

With regards to financial contagion during the covid-19 period, the GARCH BEKK model finds that USA exhibited high unidirectional linkages with the other markets, affirming its role as an important source of shock transmission across global stock markets. This can be contrasted to Fassas (2020) who finds that emerging equity markets became net transmitters of risk and risk aversion during the covid-19 period. Similar tests on bond yields show that at least eight markets (out of the twelve studied) exhibit cross-volatility transmission, supporting previous findings of the evidence of integration and co-movements in bond markets (Gilmore et al., 2010). It is also evident, especially from conditional correlations between advanced markets, that volatility becomes more pronounced during the covid-19 period.

## Declarations

### Author contribution statement

Njamba Kapalu: Performed the experiments; Contributed reagents, materials, analysis tools or data; Analyzed and interpreted the data.

Odongo Kodongo: Conceived and designed the experiments; Analyzed and interpreted the data; Wrote the paper.

### Funding statement

The authors have no funding to declare.

### Data availability statement

Data will be made available on request.

### Declaration of interests statement

The authors declare no conflict of interest.

### Additional information

No additional information is available for this paper.

## Footnotes

1 Economists postulate that the swift response of financial markets to the covid-19 outbreak can be attributed, first, to non-conventional policy interventions, such as quantitative easing and economic shutdowns (e.g., Baker et al., 2020; Zhang et al., 2020), and second, that markets exhibited unusual sensitivity to the “combined effects” of covid-19, oil price changes, the US geopolitical risk, and policy uncertainty (Sharif et al., 2020).

2 We understand financial contagion in the context of its typical usage, defined by Dornbusch et al. (2000), as a “significant increase in cross-market linkages after a shock to an individual country, or group of countries”.

3 For the AAA-rated corporate bond, Moody's Seasoned AAA Corporate Bond Yield is used as the source data.

4 https://www.bloomberg.com/opinion/articles/2020-12-01/bitcoin-is-the-tulipmania-that-refuses-to-die.

5 https://www.bloomberg.com/opinion/articles/2019-09-10/stock-market-is-acting-stranger-than-usual [Accessed 30.08.2020].

6 Impact of the COVID-19 pandemic on the Singapore Economy (mti.gov.sg) [Accessed: 07.12.2020].

7 New Zealand takes early and hard action to tackle COVID-19 (who.int) [Accessed: 07.12.2020].

8 Moody&'s downgrades South Africa&'s ratings to Ba1, maintains negative outlook - Moody&'s (moodys.com).

9 For robustness checks, we also estimate the function using OLS. Results, not reported for brevity, but available from authors upon request, are qualitatively similar to the baseline GMM results.

10 Due to space constraints, tables of outputs are not provided, but are available from the authors upon request. Rather, we report conditional correlation graphs of selected pairs to illustrate the key findings in Section 4.3.2.

11 The table of results is available from authors upon request.

12 https://www.statista.com/statistics/1102303/coronavirus-new-cases-development-russia/.

13 https://www.garda.com/crisis24/news-alerts/317771/brazil-government-confirms-first-covid-19-case-february-26-update-2.
